# Toward Ecofriendly Piezoelectric Ceramics—Reduction of Energy and Environmental Footprint from Conceptualization to Deployment

**DOI:** 10.1002/gch2.202300061

**Published:** 2023-07-08

**Authors:** Sivagnana Sundaram Anandakrishnan, Suhas Yadav, Mohadeseh Tabeshfar, Vasilii Balanov, Tharaka Kaushalya, Mikko Nelo, Jani Peräntie, Jari Juuti, Yang Bai

**Affiliations:** ^1^ Microelectronics Research Unit Faculty of Information Technology and Electrical Engineering University of Oulu Oulu FI‐90570 Finland

**Keywords:** cold sintering, DFT calculations, energy harvesting, machine learning, upside‐down composites

## Abstract

Piezoelectric materials are widely used in electromechanical coupling components including actuators, kinetic sensors, and transducers, as well as in kinetic energy harvesters that convert mechanical energy into electricity and thus can power wireless sensing networks and the Internet of Things (IoT). Because the number of deployed energy harvesting powered systems is projected to explode, the supply of piezoelectric energy harvesters is also expected to be boosted. However, despite being able to produce green electricity from the ambient environment, high‐performance piezoelectrics (i.e., piezoelectric ceramics) are energy intensive in research and manufacturing. For instance, the design of new piezoceramics relies on experimental trials, which need high process temperatures and thus cause high consumption and waste of energy. Also, the dominant element in high‐performance piezoceramics is hazardous Pb, but substituting Pb with other nonhazardous elements may lead to a compromise of performance, extending the energy payback time and imposing a question of trade‐offs between energy and environmental benefits. Meanwhile, piezoceramics are not well recycled, raising even more issues in terms of energy saving and environmental protection. This paper discusses these issues and then proposes solutions and provides perspectives to the future development of different aspects of piezoceramic research and industry.

## Graphical abstract

Piezoelectric materials are projected to grow from a niche market to broad applications in the Internet of Things (IoT) thanks to future deployment of the energy harvesting technology. Piezoelectrics still face energy and environmental challenges including high manufacturing energy consumption and the hazardous chemicals. These issues should be solved by comprehensively enhancing the ecofootprint at different stages in the piezoelectrics’ lifecycle.

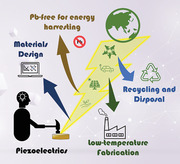

## 1 Introduction

Piezoelectricity, or the piezoelectric effect, is defined as the resultant electric displacement of a material upon being deformed (direct piezoelectric effect) and the resultant material deformation when subject to an external electric potential difference (converse piezoelectric effect). Since its discovery in 1880 and the first reports of a strong piezoelectric effect from BaTiO_3_ (BT) and Pb(Zr, Ti)O_3_ (PZT), the role of piezoelectric materials has been interesting. On the one hand, piezoelectric materials are used in a broad range of industries, such as jet nozzles in the automotive and printing sectors, ultrasonic components in medicine, and accelerometers and gyroscopes in sensing systems, to name but a few. It is their versatile electromechanical coupling capability that makes piezoelectric materials needed almost everywhere in the modern world, where mechanical and electronic devices are typically merged.

Conventional piezoelectric materials include those in the form of single crystals, polycrystalline ceramics, polymers, and composites. Single crystals within the composition family of PMN‐PT (Pb(Mg_1/3_Nb_2/3_)O_3_‐PbTiO_3_) are by far the best‐performing piezoelectric materials. For instance, the Sm‐doped PMN‐PT single crystals can achieve record piezoelectric coefficients and electromechanical coupling efficiencies among all known piezoelectrics.^[^ ^1^ ^]^ The types of excellent piezoelectric ceramics (piezoceramics) are more diverse, although with a weaker piezoelectric response than their single crystal counterparts. For instance, the Sm‐doped PMN‐PT ceramics still crowns the piezoelectric coefficients and electromechanical coupling capabilities in research,^[^ ^2^ ^]^ but the Pb(Zr,Ti)O_3_ family prevails when it comes to commercially industrial applications due to the broad tunability of their properties via soft and hard types of doping.^[^ ^3^ ^]^ Other ceramics, including BaTiO_3_‐based, (K,Na)NbO_3_‐based, and (Bi,Na)TiO_3_‐based materials,^[^ ^4^ ^]^ are also widely researched piezoelectrics which will be discussed in detail in this paper. Most piezoelectric polymers are PVDF (polyvinylidene fluoride) and its co‐polymers.^[^ ^5^ ^]^ They usually have inferior piezoelectric responses compared to single crystals and ceramics, but they are advantageous in terms of their flexibility which can tolerate material deformation to a large extent. Piezoelectric composites are formed between a piezoelectric filler (e.g., piezoceramic particles or pillars) and a piezoelectric or nonpiezoelectric matrix,^[^ ^6^ ^]^ so that the piezoelectric properties and mechanical tolerance can be balanced.

The development of piezoelectric materials faces issues such as high energy consumption and less environmental friendliness compared to other electronic materials. This paper discusses challenges and potential solutions for this aspect.

### 1.1 The Working Mechanism of Ferroelectric‐Based Piezoelectric Materials—Versatile Electromechanical Coupling

**Figure 1:**
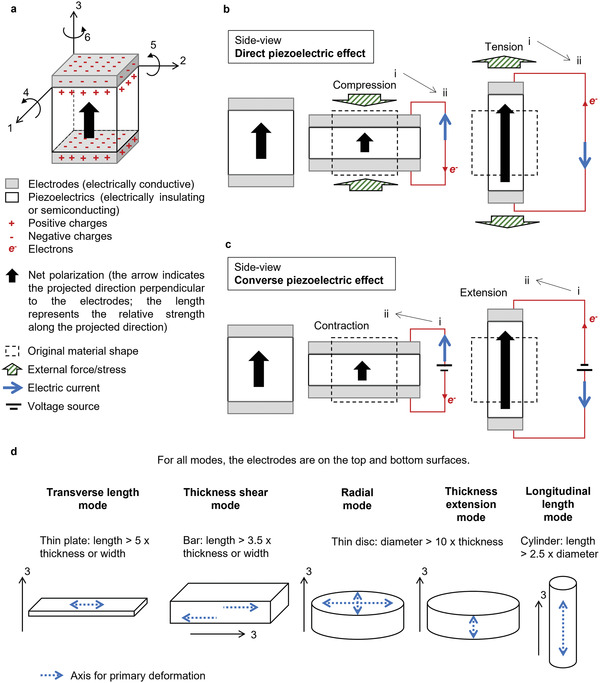
Schematics of a) definition of directions in piezoelectrics made from poled ferroelectrics, b,c) generic understanding of ferroelectric‐based piezoelectricity, and d) standardized electromechanical coupling in practice.

Modern piezoelectrics that exhibit strong piezoelectricity and thus superior feasibility in practical applications are founded on ferroelectric materials.^[^ ^7^ ^]^ Spontaneous polarizations, i.e., permanent dipoles with separated positive and negative charge centers, exist in ferroelectric materials. Furthermore, the orientations of these spontaneous polarizations can be switched by an external electric field. Thus, ferroelectrics can be “poled” by applying an external electric field, meaning that the spontaneous polarizations are forced to align. When the electric field is removed, most switched polarizations retain their orientation and thus the poled ferroelectric material exhibits a net polarization, as illustrated in **Figure** **1**a. The poled ferroelectric then becomes piezoelectric.

In Figure 1a, important factors are defined based on the assumption of a poled ferroelectric‐based piezoelectric material sandwiched between a pair of electrodes. Inside the material, positive and negative charge centers are separated with a net orientation perpendicular to the planes of the electrodes. The direction pointing from the negative charge centers towards the positive charge centers, inside the material, is defined as the ‘‘3’’ direction. Since the electrodes are electrically conductive, electrons are injected and then bond to the material surface hosting positive charge centers and in a similar way, injected positive charge carriers (i.e., in practice leftover holes after electrons have been extracted) are bonded to the surface hosting negative charge centers. In principle, the materials may be insulating or semiconducting. Such a relatively low level of electrical conductivity allows the surfaces charges to remain. Correspondingly, the other two directions in the orthogonal coordinate system are then defined as the ‘‘1’’ and ‘‘2’’ directions. In addition, the anticlockwise rotations around axes 1, 2, and 3 are defined as directions ‘‘4,’’ ‘‘5’’ and ‘‘6,’’ respectively (Figure 1a).

Figure 1b,c visualizes generic cases of direct and converse piezoelectric effects based on such polar materials, respectively. In Figure 1b (the direct piezoelectric effect), once an external compressive force is applied and the material is squeezed from its original shape, an electric potential difference is generated and, if the external circuit is closed, an instantaneous short‐circuit current flowing from the positively charged electrode to the negatively charged electrode is induced. This is because the squeezed material deformation partially misaligns the polarization vector, causing a decreased strength of the polarization projected to the ‘‘3’’ direction and thus expelling electrons out of the negatively charged electrode to compensate the change. The opposite case occurs when an external tensile force is applied, and the material is elongated. In Figure 1c (the converse piezoelectric effect), an external electric field is first applied and thus electrons are injected to the negatively charged electrode. The increased surface charge density then forces the polarization vector to align even better with the ‘‘3’’ direction as the material responds to elongate along the ‘‘3’’ direction. Similarly, in the opposite case the material contracts to respond to the decreased surface charge density which results in a partial misalignment of the polarization vector along the ‘‘3’’ direction due to an external electric field applied in an opposite direction.

Equations 1 and 2 are the generic expressions of the direct and converse piezoelectric effects, respectively, where *X* is electric displacement, *S* is strain of material deformation, *δ* is applied external stress, *E* is applied external electric field, *d* is piezoelectric charge coefficient, *ε* is permittivity, and *s* is the compliance constant. *d*, *ε* and *s* are inherent material properties but *ε* and *s* can be affected by the boundary conditions that the material is experiencing. For instance, *ε* ^*δ*^ represents the permittivity measured with a free mechanical boundary (nothing restricts the change of strain) while *s* ^E^ represents the compliance constant measured in a short circuit.

(1) $$X=d\cdot \delta +\varepsilon^{\delta }\cdot E$$

(2) $$S=s^{E}\cdot \delta +d\cdot E$$

However, Figure 1b,c only reflects how piezoelectricity is subjectively perceived in an extremely simplified format. The IEEE (Institute of Electrical and Electronics Engineers) Standard on Piezoelectricity is a representative example that clearly explains how versatile a piece of solid‐state, monolithic piezoelectric material can be when different electromechanical coupling directions are engaged.^[^ ^8^ ^]^ A set of more complex but practical electromechanical coupling mechanisms is indicated in Figure 1d. It can be clearly seen that depending on the orientation of the ‘‘3’’ direction as well as on the shape of the material and the relative position of electrodes, there are multiple types of electromechanical coupling. Equations (3), (4), (5), (6), (7), (8), (9), (10), (11) may be used to comprehensively express the direct and converse piezoelectric effects when considering specific coupling directions.

(3) $$X_{1}=d_{15}\cdot \delta_{5}+\varepsilon_{11}^{\delta }\cdot E_{1}$$

(4) $$S_{1}=s_{11}^{E}\cdot \delta_{1}+s_{12}^{E}\cdot \delta_{2}+s_{13}^{E}\cdot \delta_{3}+d_{31}\cdot E_{3}$$

(5) $$X_{2}=d_{15}\cdot \delta_{4}+\varepsilon_{11}^{\delta }\cdot E_{2}$$

(6) $$S_{2}=s_{11}^{E}\cdot \delta_{2}+s_{12}^{E}\cdot \delta_{1}+s_{13}^{E}\cdot \delta_{3}+d_{31}\cdot E_{3}$$

(7) $$X_{3}=d_{31}\cdot \left(\delta_{1}+\delta_{2}\right)+d_{33}\cdot \delta_{3}+\varepsilon_{33}^{\delta }\cdot E_{3}$$

(8) $$S_{3}=s_{13}^{E}\cdot \left(\delta_{1}+\delta_{2}\right)+s_{33}^{E}\cdot \delta_{3}+d_{33}\cdot E_{3}$$

(9) $$S_{4}=s_{44}^{E}\cdot \delta_{4}+d_{15}\cdot E_{2}$$

(10) $$S_{5}=s_{44}^{E}\cdot \delta_{5}+d_{15}\cdot E_{1}$$

(11) $$S_{6}=s_{66}^{E}\cdot \delta_{6}$$

For parameters *X*, *S*, *δ*, and *E*, the subscript defines the direction of the material response (Figure 1a). For *d*, *ε*, and *s*, the first subscript defines the direction of the material response whilst the second subscript defines the direction of the corresponding input (Figure 1a). For instance, *d*_31_ means the piezoelectric charge coefficient measured when an external stress is applied in the ‘‘1’’ direction and then the charge displacement is induced in the ‘‘3’’ direction (planes of electrodes perpendicular to the ‘‘3’’ direction). As mentioned above, it is such a multidimensional interaction between mechanical and electrical energies that makes piezoelectric materials widely used in both research and industry.

### 1.2 Development Potential of the Research on Piezoelectrics—Why Does It Matter for Global Energy and Environmental Issues?

**Figure 2:**
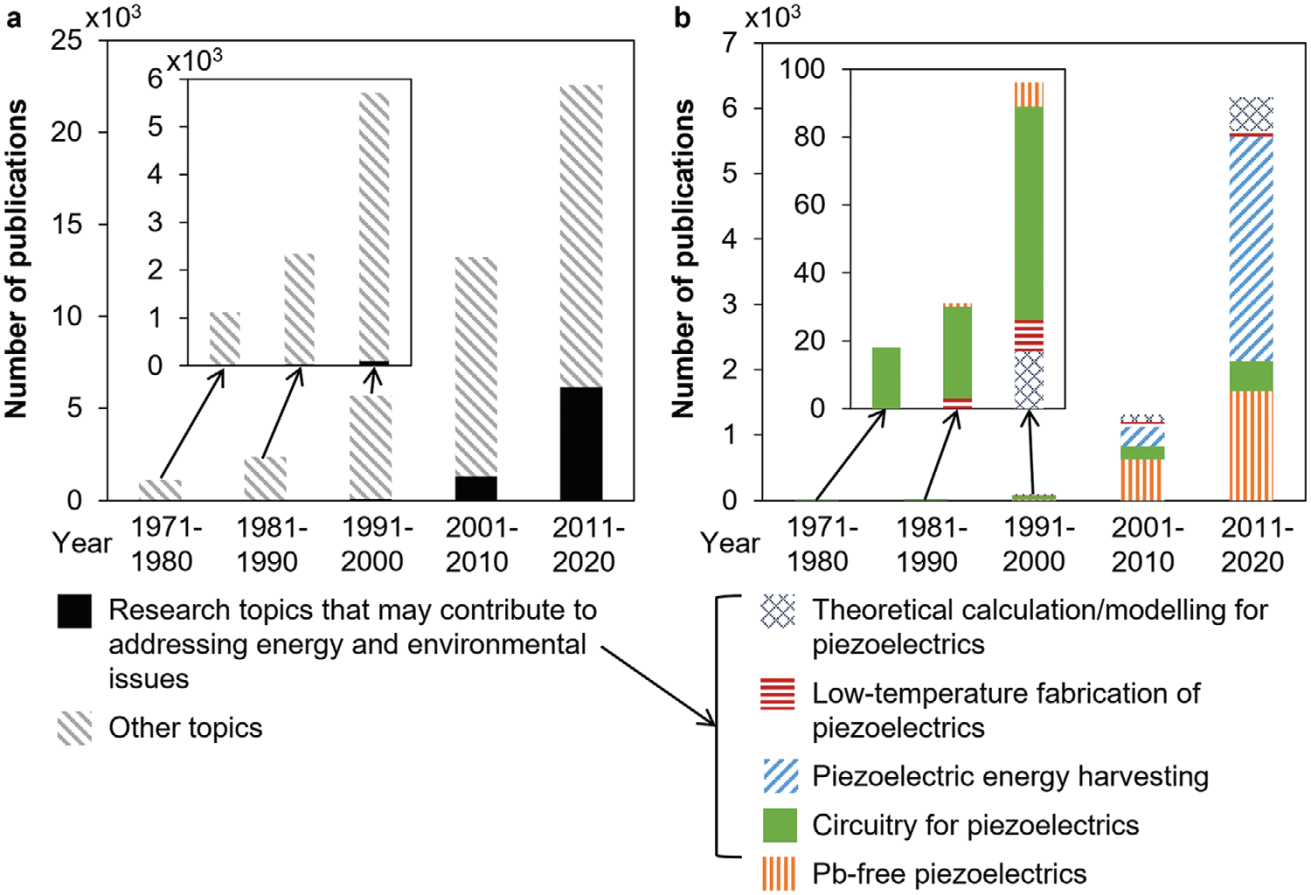
Number of publications in the recent five decades for a) total piezoelectric works and those which may contribute to addressing energy and environmental issues, and b) breakdowns of the research topics relevant to energy and environmental issues. Web of Science was used as the literature searching database.

Contrary to the broad area of applications, piezoelectric components have been only a niche market in the past decades in terms of quantity, compared to other electronics such as Si‐based components (e.g., diodes and transistors), dielectrics (e.g., capacitors) and electromagnetic modules (e.g., generators and resonators). This undermined role of piezoelectrics does not accord with their great capability. Nevertheless, the situation of piezoelectrics has been rapidly changing in the past decades. **Figure** **2**a shows the evolution of the number of publications for works on piezoelectrics, including theories, materials, devices, circuits, and applications in the past five decades. The number in each decade almost doubled that of the previous decade, with over 20 thousand published works for piezoelectrics in the 2010s, from just one thousand in the 1970s. Notably, energy and environmental issues were not paid any attention in the 1970s and 1980s, while from the 1990s research topics that may contribute to addressing these issues emerged and then have developed significantly in the 2000s and 2010s. In particular, nearly 50% of the growth in the number of publications for piezoelectrics in the 2010s were driven by topics relevant to energy and environmental issues.

Indeed, society has realized potential global challenges including possible energy and environmental crises since roughly the 1990s and the piezoelectric research community has followed at the same pace. This article aims to revisit representative piezoelectric works from the aspect of helping to address these issues. By reviewing such works, concerns over the development of piezoelectric materials in the future world, with an exploding capacity of smart electronics but under energy and environmental pressures, are raised. Meanwhile, perspectives toward solving and avoiding corresponding problems are provided.

The rationale of revisiting piezoelectric research from the energy and environmental aspect is visible in Figure 2b and **Table** **1**. It can be seen in Figure 2b that since the 2000s, piezoelectric energy harvesting has become a dominant topic among all energy‐related piezoelectric research. Piezoelectric energy harvesting means that by taking advantage of the direct piezoelectric effect (see Figure 1b), ambient kinetic energy such as vibration, acceleration, impact, etc. is collected and converted to electrical energy. Therefore, piezoelectric energy harvesters may be used to scavenge waste environmental kinetic energy and then used to power smart sensors in wireless sensor networks (WSN). For instance, in the machinery industry abundant mechanical energy is wasted amid unwanted machinery vibrations. At the same time, an excess level of vibration can be detrimental to the reliability and lifetime of machines. Self‐sufficient vibration monitoring devices powered by piezoelectric energy harvesters may be a case of an ideal use by circulating the mechanical‐electrical energy flow and thus, reusing the energy which would otherwise be wasted, promoting energy‐efficient design.^[^ ^9^, ^10^ ^]^

**Table 1:** Summary of features of the piezoelectric research topics related to energy and environmental issues

Stage in the material/device lifecycle	Energy issue	Potential solution	Expected outcome	Research needed
Materials design	Waste of energy caused by experimental trial and error	Prediction of piezoelectric properties by calculation/simulation	Energy saving from unnecessary experiments	Reliable and universal models for different types of piezoelectric materials
Manufacturing	High energy consumption during high‐temperature treatment	Low‐temperature fabrication of high‐performance piezoelectrics	Energy saving from heating and firing	Fabrication methods of robust and durable piezoelectrics with minimized heat treatment process
Deployment (early)	Long energy payback time	Piezoelectric energy harvesting	Energy saving and/or generation during usage	Development of efficient and highly capable energy harvesters
Deployment (late)	Waste of energy during maintenance of nonautonomous systems	Versatile circuitry for self‐sufficient electronics	Energy saving during usage	Creative and case‐by‐case piezoelectric energy harvesting systems
Recycling	Vanishing of initial energy investment resulting in large energy footprint	Giving a second life to old and disposed piezoelectric components	Reduction of demand in initial energy investment thus smaller footprint	Proper recycling methods that can retain initial piezoelectric properties
Trade‐off between energy and environmental issues	Lower performance/energy consumption ratio of Pb‐free piezoelectrics compared to Pb‐containing counterparts	High‐performance Pb‐free piezoelectrics for energy harvesting	Balanced ecological interrelationship in a larger picture	Engineering of Pb‐free piezoelectrics for energy harvesting applications

That is to say, the rapid growth of piezoelectric energy harvesting has been driven by the promise of the Internet of Things (IoT) which will supply billions to trillions of sensors and WSN to the smart society. For instance, there have been 6.4 billion connected sensors and other IoT devices globally in 2021, according to the statistics provided by the IEA (International Energy Agency).^[^ ^11^ ^]^ To be powered by energy harvesting technology, the WSN will be expected to drastically reduce their reliance on batteries as a power source which have the drawbacks of limited capacity and short lifespan. In comparison, energy harvesters are in principle infinite power sources. Using batteries as a power source for electronics may also raise environmental concerns due to the demanding mining of toxic elements (e.g., Pb, Cd, Ni) as well as when dead and disposed of batteries are not properly recycled (i.e., toxic elements being released to the environment). Looking at a wider scenario, digital technologies and data centers were estimated to consume around 1% of the global electricity demand in 2019, while this number could go up to 5% by 2025 and then to 20% by 2040.^[^ ^11^ ^]^ For this reason, it is worth admitting that piezoelectric energy harvesting, together with the general energy harvesting technologies, may play a non‐negligible role in either causing or solving a possible energy crisis in the future.

### 1.3 Challenges of Piezoelectric Research—The Authors’ Point of View and the Structure of This Article

However, as indicated by Table 1, piezoelectric energy harvesters have their own problems as an enabler of a green and self‐sufficient power source. Piezoelectric energy harvesters’ position in the entire lifecycle of piezoelectric materials is thought to be at the early deployment stage in devices, which are yet to be embedded in complete and functional electronic systems. The major issue of current piezoelectric energy harvesters is their long energy payback time as an electric generator. Such a problem is not only in the functionality of the harvester itself but also originates from the manufacturing stage (Table 1). Taking a rough estimation, a cm^3^‐sized, relatively high‐performance piezoelectric energy harvester may produce µW to mW‐level power depending on the working conditions. The piezoelectric materials that the harvesters are made from are mostly ceramics which need to be fabricated at high temperatures (e.g., >1000 °C). A furnace providing such a heat typically consumes 10 kW power for an effective chamber size of 4500 cm^3^ (e.g., a muffle furnace with the maximum temperature of 1600 °C). Assuming such a furnace may host 4000–5000 (this is already an extraordinary number in practice) harvesters at a time and a typical heat treatment lasts two hours, the consumed energy needs half a year in the best case but hundreds of years in the worst case to be paid back by all manufactured harvesters of this batch in service at the same time. This is yet to count the energy to be consumed by the mining of raw materials, powder synthesis, and postprocessing procedures. Undoubtedly, energy saving enabled by possibly low‐temperature fabrication is necessary during the manufacturing stage.

The need for energy saving further extends to the materials design stage (Table 1). High‐performance piezoelectric energy harvesters need new piezoelectric materials with superior piezoelectric properties. Unfortunately, the history of the development of new piezoelectrics has overwhelmingly been based on experimental trial and error. This means that high‐temperature, energy‐consuming treatment is also used frequently in the laboratory. The community lacks reliable models that are able to calculate and predict piezoelectric properties without the need for trial‐and‐error experiments. It can be seen in Figure 2b that the amount of research on both theoretical calculation/modeling and low‐temperature fabrication of piezoelectrics is considerably smaller compared to those on piezoelectric energy harvesting. They need to pick up the pace and, therefore, Sections 2 and 4 of this article discuss these two aspects, respectively.

While energy saving from the materials design and manufacturing stages may be achieved in absolute numbers, nominal and equivalent energy saving in a relative sense may also be realized in the deployment stages. For instance, high efficiency and large energy harvesting figures of merit of the piezoelectrics used in the harvesters are likely to result in a better energy payback expectation. Meanwhile, as can be seen in Figure 2b, environmentally friendly, Pb‐free piezoelectrics are another important topic. Although environmental issues are not necessarily correlated to energy issues, in the case of piezoelectric energy harvesting, Pb‐free piezoelectrics may become a tricky point especially when the trade‐off between energy saving and generation is to be balanced. For such a reason, Section 3 stresses on the aspect of energy harvesting performance versus environmental/energy benefits when developing Pb‐free piezoelectrics. The discussions in Section 3, where potentially the largest energy and environmental benefits may originate, lay the foundation for reduction of an ecological footprint for not only energy harvesting applications but also for the entire future piezoelectrics industry. Therefore, Section 3 is the specific topic focus in this paper, taking a central position and thus delivering the core messages.

At the end of their life, piezoelectrics are less likely to be able to be recycled efficiently based on the current technologies, especially for those with Pb‐containing components (e.g., the popular PZT, widely distributed in various devices) which require special care when recycling the toxic Pb. With some recent pioneering research, however, this view is being changed. It may be possible in the future that disposed piezoelectric components can be processed with a method demanding much less energy, but which may enable the reuse of waste piezoelectrics. This would also eventually contribute to energy saving by reducing the demand for initial energy investment for producing new piezoelectrics and thus help to minimize the energy footprint. The method itself will be mostly introduced in Section 4 but its particular role on the recycling stage will be discussed in Section 5.

At the end, Section 6 provides perspectives on routes towards an energy and environmentally friendly piezoelectrics research and industry.

## 2 Materials Design Stage—Theoretical Calculation and Modeling of Piezoelectrics

As has been explained in Section 1.1, the superior piezoelectrics are ferroelectrics, among which the oxide perovskite structure proves to be the king.^[^ ^7^ ^]^ The unit cell of any ferroelectric oxide perovskite is constituted by an oxygen octahedron with a B‐site cation occupying the center. The six oxygen anions sit at the face centers of a prototype cubic structure in which eight A‐site cations occupy the corners. This is widely known as the ABO_3_ structure. Below the Curie temperature, the highly symmetrical cubic structures undergo phase transitions which induce relative shifts between the positive and negative charge centers and hence the formation of spontaneous polarization at the unit cell level. The regions containing the same orientations of unit cell polarizations are known as ferroelectric domains, and a ferroelectric material initially consists of randomly oriented domains. The domains may be switched by an external electric field greater than their coercive values and furthermore, they remain largely aligned in the same direction even if the external electric field is removed. Such a process is known as poling, which gives rise to piezoelectricity. The strong piezoelectricity exhibited by ferroelectric oxide perovskites is usually derived from high remanent polarization values (after poling) as well as high domain wall mobilities.^[^ ^4^ ^]^

Nevertheless, in practice, the combination of high remanent polarizations and high domain wall mobilities comes from complex ferroelectric solid solutions in which more than one element occupies the A‐ and/or B‐sites. Ferroelectric solid solutions pose a significant challenge due to their intricate nature, as various factors play a role in determining their properties, such as the Curie temperature, phase transitions and coercive fields which may significantly affect the resultant piezoelectricity. This section will portray the evolution of the theoretical understanding of complex ferroelectric solid solutions and the upcoming challenges to find more eco‐friendly options for high‐performance applications. The underlying goal of theoretical research has been the prediction of a material's properties from its composition and to support the experimentalists. Ferroelectric solid solutions may possess various phases and exhibit various phase transition behaviors depending on the material compositions and scales (e.g., bulk, nanoscale, thin film). This complexity makes it difficult but motivating to study them in detail.

The local structures and phases of ferroelectric oxide perovskites change with their compositions.^[^ ^12^, ^13^, ^14^ ^]^ These perovskites are primarily studied using i) first‐principles density functional theory (DFT),^[^ ^15^ ^]^ ii) molecular dynamics (MD) simulations,^[^ ^16^, ^17^ ^]^ and iii) phenomenological phase‐field models (PhFM).^[^ ^18^, ^19^ ^]^ Over the past decades, binary solid solutions have been explored and recently, the focus has been upon multicomponent ferroelectric perovskites including (1‐*x*)Pb(Mg_1/2_Ti_1/2_)O_3_‐*x*PbTiO_3_ and (1‐*x*‐*y*)Pb(Mg_1/2_Ti_1/2_)O_3_‐*y*Pb(In_1/2_Nb_1/2_)O_3_‐*x*PbTiO_3_.^[^ ^20^, ^21^, ^22^, ^23^ ^]^ Although these multicomponent systems exhibit superior piezoelectricity, their underlying relaxor nature extends their complexity as a result of an inhomogeneous composition at the sub‐nanoscale level that possesses various nanoregions with local dipole orientations.^[^ ^24^, ^25^, ^26^, ^27^, ^28^ ^]^

In the pursuit of exploring theoretical solutions to understand ferroelectric oxide perovskites, it is quintessential to elucidate i) the correlation of the composition and structure to the consequent microscopic and macroscopic properties and ii) the role of chemical and physical interactions in potentially better or worse overall material behavior.

### 2.1 Phase Diagram and Phase Transitions

**Figure 3:**
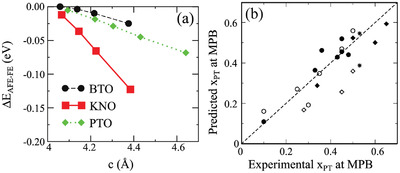
a) Energy difference between AFE and FE phases as a function of lattice parameter *c* for BTO (BaTiO_3_), KNO (KNbO_3_) and PTO (PbTiO_3_), wherein the energies have been calculated for structures with a fixed A‐site and oxygen octahedron cage. Reproduced with permission.^[^ ^31^ ^]^ Copyright 2021, John Wiley and Sons. b) Comparison of experimental and predicted MPB locations for different PbB′B″O_3_‐PbTiO_3_ and BiB′B″O_3_‐ PbTiO_3_ based solid solutions (*x*_PT_ stands for the mole fraction of PbTiO_3_). The predicted MPBs have been calculated using the formula provided in literature.^[^ ^32^ ^]^ Reproduced with permission.^[^ ^32^ ^]^ Copyright 2005, AIP Publishing.

The compositional phase diagram for a ferroelectric oxide perovskite offers information about various phases based on the change in composition and defines the possible morphotropic phase boundary (MPB) near which one usually finds the best‐performing piezoelectrics. The phase diagram also provides information on polarization vectors and on the possible antiferroelectric (AFE) to ferroelectric (FE) phase transition. It has been found that the AFE‐FE transition is based on the size of the B‐site cation. For instance, when a smaller‐sized B‐site, Sc_0.5_Nb_0.5_, is replaced with a larger one, Yb_0.5_Nb_0.5_, the PbBO_3_‐type perovskite changes from the FE state to the AFE state due to the decreased atomic displacement.^[^ ^29^ ^]^ The AFE phase needs to be avoided in piezoelectrics as it does not provide viable piezoelectricity in itself. The AFE structure is a result of the oxygen octahedron rotation which leads to a shorter displacement of cations and antiparallel Pb displacements.^[^ ^30^ ^]^ In recent DFT studies, the lower energy state has been found to be the antiparallel B‐site displacement direction when competing between being displaced in the parallel (single domain) and antiparallel (multiple domains) directions, as shown in **Figure** **3**a.^[^ ^31^ ^]^

These results have been explained using the simple argument of the minimization of electrostatic energy. In the AFE state, the electrostatic energy is reduced with the antiparallel arrangement while increasing the repulsion between the A‐site and B‐site. Cations with smaller displacement possess less repulsive force between the A‐ and B‐sites. In the case of cations with larger displacement, the repulsion between the A‐ and B‐sites is more prominent and hence the FE phase is favorable. The parallel arrangement in the FE phase reinforces the A‐site displacement and, in turn, reduces the repulsive force between A‐ and B‐sites. Therefore, the AFE‐FE phase transition in Pb‐based solid solutions can be understood as a function of the average B‐site distortion relative to the oxygen octahedron.

On the other hand, the rhombohedral to tetragonal phase transition also plays a crucial role in the piezoelectricity of ferroelectric oxide perovskites. An analytical formula has been derived by considering the interplay between the basic characteristics of the cations to predict the rhombohedral‐tetragonal MPB locations in Pb‐ and Bi‐based ferroelectric oxide perovskites.^[^ ^32^ ^]^ The formula explains how the B‐site distortions and their ionic sizes are able to affect the MPB and it has been proven useful in simple and multicomponent ferroelectric perovskite solid solutions, as shown in Figure 3b.

### 2.2 Curie Temperature

The Curie temperature (*T*_c_) is defined as the temperature above which the polar, ferroelectric state of the material changes to the non‐polar, paraelectric state, and the material loses its potential to become piezoelectric by poling. However, it should be noted that the paraelectric state of oxide perovskites or any centrosymmetric structure may still show a good electromechanical response with the help of the flexoelectric effect or ionic separation.^[^ ^33^, ^34^, ^35^ ^]^ These more recently discovered phenomena greatly broaden the future options for excellent piezoelectric materials. Here, the tentative issue may be that the induced strong piezoelectricity is highly dependent on the compositions’ and materials’ external boundary conditions. For the time being, such an issue imposes obstacles for practical applications but these new mechanisms to create high‐performance piezoelectrics should be paid significant attention in terms of their evolution of theory and development of functional properties.

Back to the ferroelectric oxide perovskites, the *T*_c_ sets the maximum operating temperature of the materials. Clearly, high‐temperature applications demand high‐*T*_c_ materials and hence it is important to understand the dependence of *T*_c_ on relevant factors in order to enable reliable prediction of new ferroelectric perovskites. Modern ferroelectric compositions are complex, containing multiple dopants and/or modifiers. Investigation on the evolution of *T*_c_ is essential for understanding the effect of dopants and modifiers causing expected as well as unexpected phase transitions. The variation of *T*_c_ with composition and external boundary conditions also provides insights into a potentially better understanding of the coercive field.

Initially, the *T*_c_ was understood to be proportional to the square of the displacement of the cations from their high‐symmetric positions.^[^ ^36^ ^]^ This understanding has been improved with the findings that the *T*_c_ may be proportional to the square of the DFT‐calculated polarization (*P*) at 0 K.^[^ ^37^, ^38^ ^]^ An even better understanding has been made by fitting the experimental *T*_c_ data with the DFT‐calculated *P* ^2^ as well as a new model‐predicted *P* ^2^ by Equation 12, where *α* and *c* are constants.

(12) $$T_{c}=\alpha P^{2}+c$$

**Figure 4:**
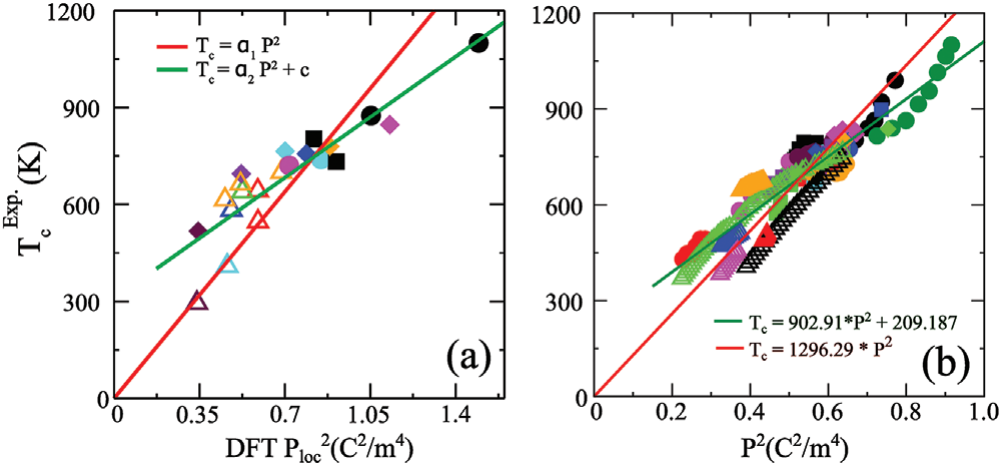
Dependence of experimental *T*_c_ on a) the DFT‐calculated *P* ^2^ with the linear fitting constants *α*_1_, *α*_2_, and c as 913.52, 535.23, and 308.43, respectively. b) A new model‐predicted *P* ^2^ for different Bi‐ and Pb‐based PTO solid solutions. The fitting constants *α*_1_, *α*_2_, and *c* are 1296.29, 902.91, and 209.19, respectively. Reproduced with permission.^[^ ^39^ ^]^ Copyright 2022, John Wiley and Sons.

**Figure** **4** depicts that Equation 12 has worked better for Pb‐ and Bi‐based perovskite oxide solid solutions.^[^ ^39^ ^]^ Despite the better fitting, Equation 12 has caused some confusion because it suggests that a material without any intrinsic polarization would still possess a ‘‘*T*_c,_’’ which is not in line with the common perception of physical rules. There must be unknown factors, which require further investigation in revised or new models.

### 2.3 Coercive Field

The coercive field (*E*_c_) reflects the minimum energy required to switch the polarizations or domains of ferroelectric materials at given frequencies. It indicates the lower limit of the applied electric field in the poling process as well as the upper limit in piezoelectric applications since an electric field higher than the *E*_c_ may degrade or even destroy the piezoelectricity by depoling the material. For instance, in a binary‐state ferroelectric memory cell, *E*_c_ determines the electric field required to switch the polarization, i.e., the threshold for rewriting into the memory. A lower *E*_c_ may benefit for faster and more energy‐efficient data storage while a higher *E*_c_ reduces the volatility of the stored data. Also, in piezoelectric actuators, a lower *E*_c_ suppresses the upper limit of the working electric field applied in the opposite direction to the polarization and thus restricts the range of actuation in the contraction mode (see Figure 1c). Therefore, *E*_c_ must be optimized to achieve optimum device performance and understanding of the evolution of *E*_c_ is crucial for designs of novel ferroelectric and piezoelectric materials and devices.

Early studies have explained that the domain switching may be proportional to the factor exp(‐*E*_act_/*E*) where *E*_act_ is the activation field.^[^ ^40^ ^]^ However, later ab initio and experimental studies have found inconsistency with the early theories and a simple analytical model has even denied the previously known models.^[^ ^41^, ^42^, ^43^, ^44^ ^]^ Recent studies have illustrated that the *E*_c_ measured at a certain temperature (*T*) may be proportional to *σ*_71_(*T*)^2^/*P*(*T*), for instance, *E*_c_(300K) ≈ (*T*_c_‐300)^1.5^ as a simple explanation where *σ*_71_ is the energy of the 71° ferroelectric domain walls.^[^ ^31^, ^45^ ^]^ This relationship has also been validated by plotting the *E*_c_ of several ferroelectric ceramics and single crystals as a function of *T*_c_ based on experimental data.^[^ ^39^ ^]^ In particular, the fit for the data of single crystals has shown a better agreement between prediction and experiment than those of ceramics where the polycrystalline microstructure tends to increase the deviation from the intrinsic *E*_c_ values of the monocrystalline structure. As piezoelectric ceramics hold a majority share in industrial applications, the above‐mentioned increased level of deviation in the prediction of *E*_c_ for ceramics implies the necessity for further development of the theoretical models.

### 2.4 Machine Learning and Multi‐Level Modeling

In recent years, machine learning (ML) techniques have started to be used for the material design of ferroelectrics. Although the complexity of the ferroelectric perovskites also applies to the ML methods, they can be a much greener and faster approach to predicting new materials compared to the conventional experimental trial and error methods (both energy and time consuming) and the DFT calculations (which may save energy but are still time consuming). There seems to be a temporary dilemma between the experimental and ML studies. On the one hand, the endless possibilities of compositional and phase variations make experimental studies tedious. On the other hand, a significant amount of diverse data from experiments are needed to properly train the ML models and the current ML studies are limited by the available data, hindering the search for new materials.

**Figure 5:**
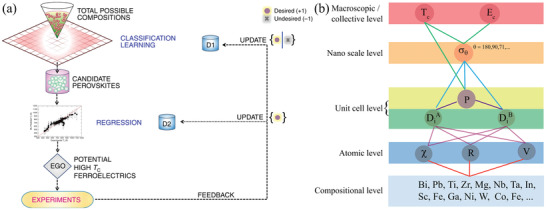
a) Schematic presentation of the two‐step machine learning model. Reproduced under terms of the CC‐BY 4.0 license.^[^ ^46^ ^]^ Copyright 2018, Springer Nature. b) Schematics of a multilevel model for predicting ferroelectric properties of Pb‐based perovskite oxide solid solutions. The atomic properties include electronegativity (*χ*), ionic radius of cations (*R*), valency of the cation (*V*), and displacement of A‐ and B‐sites (*D*_i_^A^ and *D*_i_^B^), respectively. The macroscopic properties include polarization (*P*), domain wall energies (*σ*_*θ*_), Curie Temperature (*T*_c_), and coercive field (*E*_c_). Reproduced with permission.^[^ ^39^ ^]^ Copyright 2022, John Wiley and Sons.

Some early studies have used the Pauling electronegativity, the Martynov–Batsanov electronegativity and the ratio of valence electron number to nominal charge to demonstrate an ML model that predicts the displacements of cations.^[^ ^46^ ^]^ The studies have also provided a two‐step ML method for the prediction of high *T*_c_ ferroelectric perovskites. It has been predicted that there could be as many as 60 000 possible compositions, of which only about 170 have been explored. Not all the compositions would possess the perovskite phase and hence the rational of the two‐step model. The model includes the classification of the materials with the perovskite structure and then evaluates their *T*_c_ with regression, as shown in **Figure** **5**a. Simple cation characteristics including the tolerance factor, the Mendeleev number and the valence electron of the cation that are easily available as descriptors have been used and the model has correlated them with the macroscopic properties including the *T*_c_ and the perovskite phase stability.

Meanwhile, a multilevel model has been developed for the prediction of macroscopic properties such as the polarization, coercive field and Curie temperature for Pb‐based ferroelectric solid solutions from their perovskite compositions.^[^ ^39^ ^]^ As illustrated in Figure 5b, the multiple levels accommodate different cation characteristics including the valency, electronegativity, and ionic radius for the prediction of the polarization and further target properties such as the *T*_c_ and *E*_c_. This multilevel model has described the importance of the cation displacement in the accuracy of predicting *T*_c_ and *E*_c_. It has also demonstrated success in dealing with complex and multicomponent ferroelectric perovskites.

Nevertheless, it should be realized that the actual piezoelectric properties, such as the essential piezoelectric coefficients and electromechanical coupling factors which directly determine the usability of piezoelectric materials, have not been touched by the above theoretical methods. Although there have been scattered studies on the prediction of *d*_33_, the methods are rather material specific and based on DFT which is computationally expensive.^[^ ^47^, ^48^, ^49^ ^]^ Indeed, the prediction of piezoelectric properties still faces challenges, and this will be discussed in Section 6.

## 3 Manufacturing Stage Considering Deployment—Trade‐Offs between Selections of Pb‐Containing and Pb‐Free Piezoelectrics

It can be realized in Section 2 that most current DFT and ML models are developed for Pb‐containing ferroelectric oxide perovskites. Although researchers have seen some hope of predicting new Pb‐containing ferroelectric perovskites using the models, the Pb‐free counterparts are still regrettably largely relying on experimental trial and error. Pb‐free piezoelectric materials rose to prominence primarily due to the EU's legislation excluding the use of toxic elements (including Pb) in most electronics.^[^ ^50^ ^]^ However, in the legislation, an exemption was made for Pb being used in piezoelectrics due to the versatility of Pb in these applications and very few device‐level alternatives. The exemption is still active after two decades following multiple renewal requests for the extension of the exemption's deadline submitted by leading stakeholders of piezoelectrics research and industry.^[^ ^51^, ^52^ ^]^ Therefore, research on Pb‐free piezoelectrics and their continuous optimization for device‐level manufacturing remains a growing trend in modern piezoelectrics research despite having been under investigation for almost four decades.^[^ ^53^ ^]^

Regarding the topic at hand, Pb‐free piezoelectrics have an immediate energy and environmental benefit over their Pb‐based counterparts, which is energy saving during the post‐treatment and cleaning. For Pb‐based piezoelectrics, during their mining, fabrication, and ultimate disposal, toxic Pb is usually leeched out into the surrounding environment and potentially demands an immense amount of energy for post‐treatment and cleaning.^[^ ^54^ ^]^ Nevertheless, if the Pb‐free alternatives are replacing the Pb‐based ones at a higher energy cost of fabrication, the benefits of energy harvesting by not using Pb would be diminished. The main reason the energy budget of fabrication is explored in this article, is that energy harvesting applications aim to minimize the energy wasted into the environment (hence the term “harvesting”). Usage of materials with a possibly high energy price of fabrication would be unsustainable in the deployment stage when the energy harvesting operation must pay back the excessive use of energy during the fabrication.

Therefore, this section aims to analyze promising Pb‐free piezoceramics for the application of energy harvesting, keeping in mind their energy consumption during fabrication. The goal of studying from such a fresh point of view is to compare these two parameters (i.e., the energy harvesting capability and energy consumption during fabrication) of Pb‐free alternatives with those of the commonly used Pb‐based examples (mostly PZT and its variants). This will provide readers with the knowledge regarding whether Pb‐free alternatives can replace the PZT family in the energy harvesting sector both performance‐wise as well as sustainability‐wise.

### 3.1 Important Parameters

#### 3.1.1 Piezoelectric Charge and Voltage Coefficients

Alongside the parameters introduced above, *d* (the piezoelectric charge coefficient) and *ε* (the material's permittivity), the piezoelectric voltage coefficient (*g*) is defined as *g* = *d*/*ε*. The parameter *g* relates to the electric field generated at the electrodes when a unit stress acts on the piezoelectric material. It is worth noting that the *g* values are not provided directly by most authors in the literature reporting piezoelectrics research. In this article, the cited *g* values have been manually calculated from the *d* and *ε* values found in corresponding references.

In addition, the *ε* values used for the calculation of the *g* values should have been measured from the poled piezoceramics. However, some reported *ε* values may have been measured from the unpoled ceramics, or authors have not mentioned whether the reported values are for the poled or unpoled materials. In these cases, the calculated *g* values are marked with an additional footnote (i.e., *g* ^a)^).

#### 3.1.2 Piezoelectric Energy Harvesting Figure of Merit

The piezoelectric energy harvesting figure of merit (FOM) is calculated as FOM = *d*·*g*, and it relates to the energy density that can be harvested by the piezoelectric material. To ensure that the performances of the materials from different literature are comparable, this article adopts *d*_33_ and *g*_33_ from the longitudinal length mode shown in Figure 1d, where *g*_33_ = *d*_33_/*ε*_33_ and FOM  = *d*_33_^2^/*ε*_33_.

#### 3.1.3 Electromechanical Coupling Factor

The electromechanical coupling coefficient (*k*) is a unitless constant where *k* ^2^ is defined from the fraction of the input mechanical energy that is converted to electrical energy by the piezoelectric material. The *k* values are also influenced by the coupling directions such as *d* and *ε*. In this article, *k*_33_ and *k*_p_ (planar *k*) are employed from the longitudinal length mode and the radial mode, respectively (Figure 1d).

#### 3.1.4 Transferrable Device‐Level Performance Indicators

The parameters of the converse effect piezoelectric coefficient, *g*, *k* and FOM are positive indicators of the potential device performances for piezoelectric actuators, sensors, transducers, and energy harvesters, respectively. Therefore, comparing the values of these parameters among different materials provides reliable and transferrable information to the potential device‐level comparison, given that the structures, configurations, and working conditions of the corresponding devices are kept identical.

#### 3.1.5 The Self‐Defined Thermal Energy Indicator

For a convenient and straightforward comparison, this article defines an indicator for the thermal energy used during fabrication to be Φ. It can also be referred to as the energy budget of fabrication, implying the equivalent amount of energy consumed by a typical high‐temperature furnace during any necessary heat treatment on the piezoceramics. The energy is only associated with the furnace as it is the most energy‐intensive part of the fabrication procedure.

Two types of heat treatments are typically employed in piezoceramics fabrication consecutively: calcination and sintering. Calcination refers to the heat treatment employed to form the desired piezoceramic phases from the raw powders while sintering refers to the heat treatment that enables densification and grain growth in the piezoceramics to achieve the desired piezoelectric functionality. The material is held at a peak/hold temperature for a particular period of time referred to as the holding time in both these heat treatments. In this article, the Φ value has been calculated as the product of the hold/peak temperature of the heat treatment and the holding time. In this way, the Φ value represents the energy consumed by the furnace during the fabrication stage for the piezoceramics with the necessary functionality, i.e., the energy price paid for the piezoelectric properties obtained. The factor is assigned the unit of per 1000 °C h. The heating and cooling rates during the heat treatments are neglected for the calculations as they are among common practices in the research community and thus are usually standardized. Therefore, deviations may exist in the real energy expenditure depending on these rates. The general idea behind the Φ calculation is to introduce a measure that can be compared across many compositions. In case the heat treatment profiles are not mentioned in the corresponding literature, other literature of the same or similar type of materials has been taken.

### 3.2 Popular Pb‐Free Piezoelectric Perovskite Systems and Their Piezoelectric Properties

As discussed in Section 2, the phase transitions and MPB are crucial for achieving good piezoelectric properties in Pb‐based oxide perovskites. This is also empirically true for Pb‐free piezoelectrics based on ferroelectric oxide perovskites. Herein, the influence of the phase transitions with respect to the chemical compositions on the piezoelectric properties, especially for the energy harvesting capabilities (FOM and *k* values), is discussed for prospective Pb‐free perovskite families.

#### 3.2.1 (K_0.5_Na_0.5_)NbO_3_‐Based Ceramics

K_0.5_Na_0.5_NbO_3_ (KNN) based ceramics are one of the most widely researched Pb‐free piezoceramics owing to their high *T*_c_ coupled with high *d*_33_ values.^[^ ^55^ ^]^ KNN‐based ceramics are likely to enhance their piezoelectricity at the MPB region.^[^ ^56^ ^]^ This is due to the facile polarization rotation and the vanishing of the polarization anisotropy close to the phase boundaries.^[^ ^57^ ^]^ Without additives, these phase boundaries are usually present at extreme temperatures. In order to access the high *d*_33_ close to these phase boundaries, appropriate ion substitutions and/or fabrication of binary and ternary systems have been used to shift the MPB region closer to the room temperature. This strategy is known as phase boundary construction.

In KNN‐based ceramics, there are four general phase boundaries that may be constructed approaching the room temperature.^[^ ^58^ ^]^ The rhombohedral (Rhom.) to orthorhombic (Orth.) phase boundary is naturally present at approximately ‐60 °C and when shifted to room temperature, the *d*_33_ can be increased from ≈80 pC N^−1^ up to ≈200 pC N^−1^.^[^ ^59^, ^60^, ^61^, ^62^, ^63^ ^]^ The Orth.‐tetragonal (Tetr.) phase boundary is present at around 140 °C and when shifted to room temperature, the *d*_33_ can be further improved to ≈400 pC N^−1^.^[^ ^64^, ^65^, ^66^ ^]^ The Rhom.‐Tetr. phase boundary construction consists of the simultaneous reduction of the Orth.‐Tetr. phase boundary and the promotion of the Rhom.‐Orth. phase boundary to room temperature. In this process, increasing the amount of sufficient doping tends to suppress the Orth. phase until it vanishes. The *d*_33_ shown by the KNN ceramics with this type of phase boundary construction can reach superior values of 490–570 pC N^−1^.^[^ ^56^, ^57^, ^67^, ^68^, ^69^, ^70^ ^]^ A Rhom.‐Orth.‐Tetr. phase boundary may also be designed in the KNN system in a similar way to the Rhom.‐Tetr. boundary, but it involves no suppression of the Orth. phase at room temperature. The *d*_33_ values at such a phase boundary can also be high, i.e., around 400–480 pC N^−1^.^[^ ^71^, ^72^, ^73^, ^74^ ^]^

#### 3.2.2 (Bi_0.5_Na_0.5_)TiO_3_‐Based Ceramics

Bi_0.5_Na_0.5_TiO_3_ (BNT) based ceramics are known for their large strain capabilities and large remnant polarization after saturation and thus are typically considered for use in actuator applications. Their piezoelectric performance is likely to be limited by the depolarization temperature (*T*_d_), wherein the AFE phases emerge, and the piezoelectricity vanishes. The piezoelectricity of the BNT‐based ceramics can be optimized at the phase boundaries in a similar manner to those of the KNN‐based ceramics. Typically, a Rhom.‐Tetr. phase boundary known as the MPB‐I is constructed at room temperature for this purpose, which can be done via the introduction of suitable binary and ternary solid solutions.

The BNT‐BT binary system, i.e., (1‐*x*)(Bi_0.5_Na_0.5_)TiO_3_‐*x*BaTiO_3_ with necessary doping, can produce *d*_33_ values in the range of 130–190 pC N^−1^.^[^ ^75^, ^76^, ^77^, ^78^, ^79^, ^80^ ^]^ The BNT‐BKT ((Bi_0.5_K_0.5_)TiO_3_) binary system and its family can produce *d*_33_ values in the range of 130–230 pC N^−1^.^[^ ^81^, ^82^, ^83^, ^84^, ^85^ ^]^ Other perovskite solid solutions such as KNN and SrTiO_3_ may also form binary systems with the BNT and even ternary systems with the BNT‐BT or BNT‐BKT to construct the MPB‐I at room temperature. These systems are able to produce *d*_33_ values in the range of 150–235 pC N^−1^.^[^ ^86^, ^87^, ^88^, ^89^, ^90^, ^91^, ^92^, ^93^, ^94^ ^]^

#### 3.2.3 BiFeO_3_‐Based Ceramics

The BiFeO_3_ (BF) based ceramics are typically characterized with low‐to‐moderate piezoelectric properties and very high *T*_c_. The high *T*_c_ enables these ceramics to be used for high‐temperature applications. However, they suffer from other issues including high coercive field, high leakage current, low resistivity, and a tendency to form impurity phases. All these issues make poling difficult.^[^ ^4^ ^]^ Proper tuning of BF‐based ceramics is usually required to improve their piezoelectric performance as well as to enable an efficient poling process.

Similar to the BNT material, a phase boundary of BF can be constructed at room temperature by forming binary and ternary solid solutions mostly with BT and its family. Ionic substitution may also be used to improve the piezoelectricity of the BF‐based ceramics, but the enhancement is mainly attributed to the reduction of impurity phases and leakage currents.^[^ ^4^ ^]^ The effect of ionic substitution is limited, and the resultant *d*_33_ values are in the range of only 45–50 pC N^−1^.^[^ ^95^, ^96^, ^97^ ^]^ In the BF‐BT binary systems, it is possible to construct Rhom.‐pseudocubic (Pseu.) and Rhom.‐Tetr. phase boundaries with greatly enhanced *d*_33_ values of 150–400 pC N^−1^. This enhancement is a combined effect of room temperature MPB, reduction of impure phases and leakage current, and formation of a denser microstructure with large grains.^[^ ^98^, ^99^, ^100^, ^101^, ^102^, ^103^, ^104^ ^]^ Additional perovskite solid solutions may be added to form other BF‐BT‐based ternary systems to achieve similar results of the construction of the phase boundaries and/or the formation of denser microstructure with bigger grains and hence enhancement of *d*_33_ values up to 135–325 pC N^−1^.^[^ ^4^, ^103^, ^105^, ^106^, ^107^, ^108^, ^109^ ^]^

#### 3.2.4 BT‐Based Ceramics

Although BT is the first discovered Pb‐free perovskite‐structured piezoelectric composition, the pure BT ceramics show quite a low piezoelectricity.^[^ ^110^ ^]^ Therefore, ionic site engineering (or ionic substitution) has been used to improve its piezoelectricity via phase boundary construction at room temperature.^[^ ^4^ ^]^ Three types of phase transitions exist in BT as a function of temperature; i.e., Rhom.‐Orth. at ≈‐90 °C, Orth.‐Tetr. at ≈0 °C and Tetr.‐cubic at *T*_c_ ≈ 120 °C. A tri‐critical triple point of the Rhom., Tetr. and cubic phases can be constructed in the Ba(Zr_0.2_Ti_0.8_)O_3_‐(Ba_0.7_Ca_0.3_)TiO_3_ (BZT‐BCT) systems.^[^ ^111^ ^]^ Appropriate dopants have been added to shift the Rhom.‐Orth. and Orth.‐Tetr. phase transitions to room temperature. Their coexistence with a Pseu. phase has also been observed in some BT‐based systems.^[^ ^112^ ^]^ All these phase boundaries are associated with high piezoelectric properties. Among the most studied BT‐based systems, the (Ba_(1_ *_–x_*_)_Ca*_x_*)(Ti_(1_ *_–y_*_)_Zr*_y_*)O_3_ (BCZT), (Ba_(1_ *_–x_*_)_Ca*_x_*)(Ti_(1_ *_–y_*_)_Sn*_y_*)O_3_ (BCST) and (Ba_(1_ *_–x_*_)_Ca*_x_*)(Ti_(1_ *_‐y_*_)_Hf*_y_*)O_3_ (BCHT) ceramics typically show the peak piezoelectric properties with *d*_33_ values achieving the ranges of 510–755 pC N^−1^, 570–680 pC N^−1^ and 450–550 pC N^−1^, respectively.^[^ ^111^, ^112^, ^113^, ^114^, ^115^, ^116^, ^117^, ^118^, ^119^, ^120^, ^121^, ^122^, ^123^, ^124^, ^125^, ^126^, ^127^, ^128^, ^129^, ^130^, ^131^, ^132^, ^133^ ^]^

#### 3.2.5 Comparison of the Piezoelectric Properties among Different Systems

**Table** **2** lists the *d*_33_, *g*_33_, FOM, *k*_p_ and Φ values of representative Pb‐free ceramics which show the optimum FOM as well as relatively low energy budget of fabrication within each family. The same parameters of commercially used PZT piezoceramics are also listed for comparison, including the PZT‐5H (soft‐PZT), PZT‐5A (PZT with good temperature stability), and PZT‐4 and PZT‐8 (two variants of hard‐PZT).^[^ ^3^, ^134^ ^]^

**Table 2:** Comparison of piezoelectric properties and required energy budget of fabrication for representative Pb‐based and Pb‐free piezoceramics

Composition	*d*_33_ [pC N^−1^]	*g*_33_ [mVm N^−1^]	FOM [x10^−12^ m^2^ N^−1^]	*k*_p_	Φ [x10^3^ °C h]	Refs.
0.96(K_0.48_Na_0.52_)(Nb_0.95_Sb_0.05_)O_3_‐0.04Bi_0.5_(Na_0.82_K_0.18_)_0.5_ZrO_3_	490	25^a)^	12.3	0.48	8.36	[69]
0.965(K_0.48_Na_0.52_)(Nb_0.95_Sb_0.05_)O_3_‐0.035Bi_0.5_(Na_0.82_K_0.18_)_0.5_HfO_3_	525	24^a)^	12.5	–	8.37	[57]
0.964(K_0.4_Na_0.6_)(Nb_0.955_Sb_0.045_)O_3_‐0.006BiFeO_3_‐0.03Bi_0.5_Na_0.5_ZrO_3_	550	24^a)^	12.9	0.53	8.36	[56]
0.96(K_0.5_Na_0.5_)(Nb_0.96_Sb_0.04_)O_3_‐0.01SrZrO_3_‐0.03Bi_0.5_Na_0.5_HfO_3_	470	27	12.5	0.51	8.40	[73]
0.96(K_0.48_Na_0.52_)(Nb_0.95_Sb_0.05_)O_3_‐0.04Bi_0.5_(Na_0.82_K_0.18_)_0.5_ZrO_3_	490	24^a)^	11.8	0.48	8.55	[67]
0.93Bi_0.5_Na_0.5_TiO_3_‐0.07BaTiO_3_	134	76^a)^	10.1	–	8.70	[75]
Bi_0.5_(Na_0.7_K_0.2_Li_0.1_)_0.5_TiO_3_	231	22^a)^	5.1	0.37	3.90	[85]
0.852Bi_0.5_(Na_0.9_Li_0.1_)_0.5_TiO_3_‐0.11Bi_0.5_K_0.5_TiO_3_‐0.038BaTiO_3_	235	26^a)^	6.1	0.30	4.06	[94]
0.7Bi_0.5_Na_0.5_TiO_3_‐0.2Bi_0.5_K_0.5_TiO_3_‐0.1Bi_0.5_Li_0.5_TiO_3_	231	22	5.0	0.37	3.95	[90]
0.865Bi_0.5_Na_0.5_TiO_3_‐0.06BaTiO_3_‐0.075Bi_0.5_Li_0.5_TiO_3_	208	23	4.8	0.37	3.90	[92]
0.7Bi_1.05_FeO_3.075_‐0.3BaTiO_3_	180	31	5.6	–	4.40	[102]
0.7Bi_1.05_Fe_0.97_Sc_0.03_O_3_‐0.3BaTiO_3_	180	34	6.2	–	4.43	[104]
0.67Bi_1.05_FeO_3_‐0.33BaTiO_3_	240	27	6.5	0.47	4.34	[103]
0.67Bi_1.05_(Fe_0.97_Ga_0.03_)O_3_‐0.33BaTiO_3_	402	45	18.3	0.32	4.43	[103]
0.970.67Bi_1.05_‐FeO_3_‐0.33BaTiO_3_‐0.03Bi_1.05_(Zn_0.5_Ti_0.5_)O_3_	324	73	23.7	0.35	4.37	[103]
(Ba_0.85_Ca_0.15_)(Zr_0.1_Ti_0.9_)O_3_	637	29	18.5	0.60	5.70	[133]
(Ba_0.85_Ca_0.15_)(Zr_0.1_Ti_0.9_)O_3_	650	16	10.6	0.53	5.68	[113]
(Ba_0.85_Ca_0.15_)(Zr_0.1_Ti_0.9_)O_3_	620	23^a)^	14.2	‐	8.70	[111]
PZT‐5H	590	20	11.8	0.68	8.60	[3]
PZT‐5A	375	25	9.4	0.63	8.60	[3]
PZT‐4	290	25	7.3	0.60	8.60	[3]
PZT‐8	225	25	5.6	0.50	8.60	[3]

^a)^ The *ε* values are given only for the unpoled ceramics, or the possible poling procedure is unknown.

From Table 2, it can be inferred that relatively high *g*_33_ values are observed for all Pb‐free piezoceramic families comparable to the PZT variants. This provides a good incentive to replace Pb‐based piezoceramics in sensor applications with Pb‐free counterparts as *g*_33_ is the figure of merit for sensors.^[^ ^135^ ^]^

Notably, the highest *g*_33_ values are seen for the representative BF‐based piezoceramics owing to their moderate *d*_33_ and low permittivity. Moreover, the BF‐based piezoceramics have a higher *T*_c_ and a lower Φ compared to the PZTs which is attractive for the sustainable production of high‐temperature sensor materials.

For actuation, the *d*_33_ measured via the converse piezoelectric effect (usually referred to as *d*_33_* and with the unit of pm V^−1^) is considered as the figure of merit. Although the *d*_33_* value has not been considered as a part of this review due to the focus being mainly on energy harvesting via the direct piezoelectric effect, the BNT‐based piezoceramics show the most promise in this aspect. Superior actuating performance with *d*_33_* values in the range of 500–1100 pm V^−1^ have been reported for these piezoceramics which are able to compete with conventional Pb‐based counterparts.^[^ ^136^, ^137^, ^138^, ^139^, ^140^, ^141^ ^]^

It should be noted that the observed *d*_33_ value is the lowest in the BNT‐based piezoceramic family since their chemistry enables them to have higher actuation properties rather than high *d*_33_ via the utilization of a different phase boundary.^[^ ^4^ ^]^

Last, transducers are also an important piezoelectric application area. The figure of merit for transducers is mainly *k*. From Table 2, it is seen that the *k*_p_ of all the Pb‐free families is generally lower than the PZT variants, making the incentive to replace the PZT family in transducers low. A lower *k* value could result in excessive heating of the piezoelectric component when working in the resonant mode together with a lower mechanical‐electrical energy conversion efficiency. Thus, the trade‐offs between the *k* values and the environmental benefits will be discussed in the context of energy harvesting in the following section.

### 3.3 Trade‐Offs between the Energy Harvesting Capabilities and Energy/Environmental Benefits

**Figure 6:**
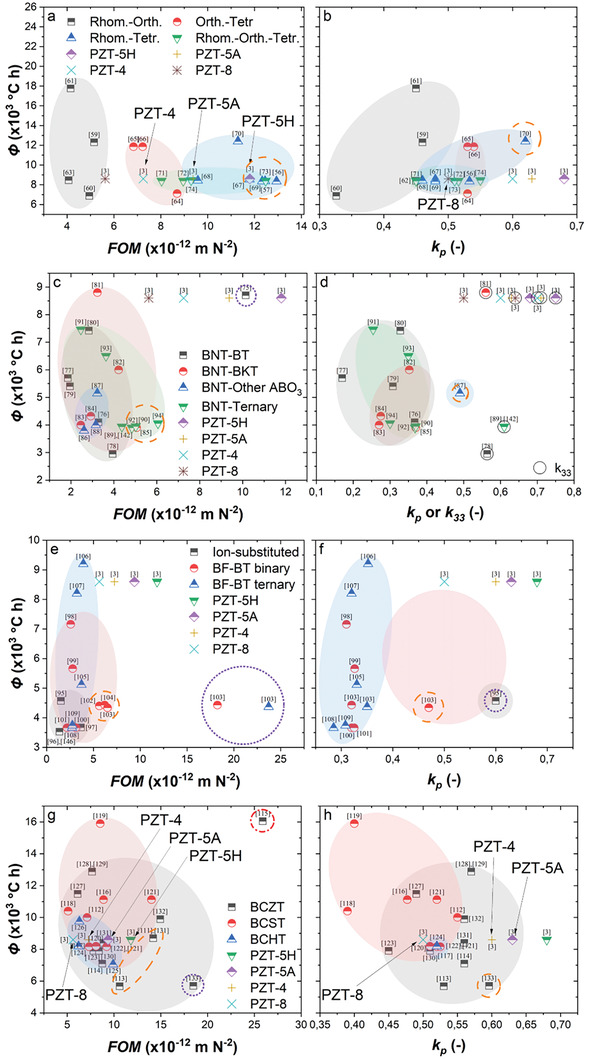
Comparison of the energy budget for fabrication with a,c,e,g) piezoelectric energy harvesting figure of merit and b,d,f,h) electromechanical coupling factor for a,b) KNN‐based, c,d) BNT‐based, e,f) BF‐based and g,h) BT‐based piezoceramics, where the positions of the PZT compositions are also marked in each subfigure. In (d), the black circles with solid lines and without shade fills surrounding certain data points mean that these data are for *k*_33_. The data are extracted from various references listed throughout this section, especially from Table 2.^[^ ^3^, ^56^, ^57^, ^59^, ^60^, ^61^, ^62^, ^63^, ^64^, ^65^, ^66^, ^67^, ^68^, ^69^, ^70^, ^71^, ^72^, ^73^, ^74^, ^75^, ^76^, ^77^, ^78^, ^79^, ^80^, ^81^, ^82^, ^83^, ^84^, ^85^, ^86^, ^87^, ^88^, ^89^, ^90^, ^91^, ^92^, ^93^, ^94^, ^95^, ^96^, ^97^, ^98^, ^99^, ^100^, ^101^, ^102^, ^103^, ^104^, ^105^, ^106^, ^107^, ^108^, ^109^, ^111^, ^112^, ^113^, ^114^, ^115^, ^116^, ^117^, ^118^, ^119^, ^120^, ^121^, ^122^, ^123^, ^124^, ^125^, ^126^, ^127^, ^128^, ^129^, ^130^, ^131^, ^132^, ^133^, ^142^, ^143^ ^]^

**Figure** **6** plots the FOM and *k* values with estimated Φ values for the selected KNN‐based, BNT‐based, BF‐based and BT‐based ceramics in comparison with the PZT family. In Figure 6a, it can be seen that the part of the KNN‐based piezoceramics constructed with the Rhom.‐Tetr. and the Rhom.‐Orth.‐Tetr. phase boundaries are the only ones that seem comparable to PZT‐5H in terms of the FOM values (as highlighted within the dashed orange circle). The high energy harvesting performance of these KNN‐based piezoceramics is attributed to the high *d*_33_ values achieved at the MPB regions where more facile polarization rotation is enabled in comparison to those of other phase boundaries.^[^ ^4^ ^]^

Meanwhile, the energy budgets required for their fabrication are mostly comparable to that of the PZT‐5H, weighing up the sustainability aspect of substituting the PZT‐5H with these KNN‐based materials.

In Figure 6b, it can be realized that the *k* values of the KNN‐based ceramics are mostly lower than those of the PZT‐5H but are comparable to those of the PZT‐8 which seems to be the bottom line of the performance in the PZT family in this aspect. The known record *k*_p_ of 0.62 has been achieved by the KNNS‐BZ‐BKH (0.95[K_0.575_Na_0.425_][Nb_0.965_Sb_0.035_]O_3_‐0.02BaZrO_3_‐0.03[Bi_0.5_K_0.5_]HfO_3_) composition (as highlighted within the dashed orange circle) which is comparable to that of the PZT‐5A.^[^ ^70^ ^]^

As mentioned above, lower *k* values lead to lower energy conversion efficiencies and/or cause higher levels of undesirable heating and thus waste energy when deployed in energy harvesters. Moreover, high levels of heating raise the temperature of the material which compromises piezoelectric properties when approaching the *T*_c_. The generally lower *k* values in KNN‐based piezoceramics may be attributed to the inherently higher dielectric losses (≈0.05).^[^ ^4^ ^]^

According to Figure 6c, all the BNT‐based ceramics have a smaller energy footprint during fabrication than the PZT family. However, it is also noted that the FOM values are much lower due to their lower *d* values. For example, the highest *d*_33_ seen amongst the BNT‐based compositions has been about 235 pC N^−1^, less than half of that for the PZT‐5H (590 pC N^−1^). The best‐performing BNT‐based piezoceramics (highlighted with the dashed orange circle), i.e., a part of the BNT ternary and BNT‐BKT binary systems, generally exhibit FOM values comparable to those of the PZT‐8. An outlier (highlighted with the dotted violet circle) is seen for the BNT‐BT system which shows a comparable energy budget and higher energy harvesting performance in comparison to the PZT‐5A.^[^ ^75^ ^]^

The relatively high energy harvesting performance is attributed to the high *g*_33_ values due to the very low *ε* values of this composition. Despite the better performance, the energy budget is also higher for this BNT‐BT system when compared to the other BNT‐based counterparts because the fabrication requires the calcination of the BNT and BT separately via a precursor‐induced method.

Similarly, from Figure 6d, it can be understood that the BNT‐based composition with the highest *k*_p_ (highlighted with the dashed orange circle) is comparable only to the worst‐performing PZT‐8 in the PZT family (*k*_p_ ≈ 0.5).^[^ ^87^ ^]^ The *k*_33_ values are even smaller and, in this sense, none of the BNT‐based piezoceramics seem to be more favorable than the PZT family.

In Figure 6e, most of the BF‐based piezoceramics are also seen to perform poorly in terms of their energy harvesting capabilities. Some of them show comparable FOM values to that of the PZT‐8 (highlighted with the dashed orange circle) and a favorable energy footprint compared to the PZT family. However, better performing outliers are found within the binary and ternary BF‐BT systems which exhibit superior energy harvesting capabilities (highlighted with the dotted violet circle) even compared to the PZT‐5H.^[^ ^103^ ^]^

Surprisingly, they also need much smaller energy budgets. Such favored features have been attributed to the addition of super‐tetragonal elements/ceramics into the unit cells, namely gallium in the BF‐BT binary system and Bi_1.05_(Zn_0.5_Ti_0.5_)O_3_ in the BF‐BT ternary system, that have increased the *d*_33_ values to a large extent. Furthermore, excess Bi has been introduced during the fabrication, which has avoided the detrimental effects of Bi loss due to evaporation at high temperatures.

Figure 6f reveals that most BF‐based ceramics produce vastly inferior *k*_p_ compared to the PZT family. The best‐performing BF‐based composition shows a *k*_p_ value of ≈0.6 (highlighted with the dotted violet circle), comparable to that of the PZT‐4.^[^ ^95^ ^]^ Regrettably, the FOM of such a composition is too inferior to be significant (Figure 6e). If both the FOM and *k* values are jointly considered, the favorable one is a BF‐BT binary system (highlighted with the dashed orange circle) of which the *k*_p_ value is comparable to that of the PZT‐8.^[^ ^103^ ^]^

Lastly, for the BT‐based ceramics, it can be seen in Figure 6g that high energy harvesting performance is shown predominantly by the BCZT system, followed by the BCST and BCHT systems. However, only the BCZT compositions seem to have superior FOM values as well as lower or similar Φ values compared to the PZT‐5H (highlighted with the dashed orange circle). It is noted that despite the high *d*_33_, the ultra‐high permittivity of the BT‐based ceramics hinders their development in terms of *g*_33_ and FOM. Among all the BT‐based ceramics, an outlier exists (highlighted with the dash‐dotted red circle) corresponding to a BCZT composition with very high FOM but also high Φ values.^[^ ^115^ ^]^ This is attributed to employ the texturing process which is known as the TGG (templated grain growth) method employed. The method involves using aligned single crystal templates for nucleation and growth of the crystallites, resulting in an increased fraction of oriented crystallites. The textured microstructure and hence the decreased randomness of the crystalline orientation has increased the *d*_33_ tremendously, not only due to the reduction of polarization anisotropy but also the involved heat treatment at high temperatures for long periods of time to increase the fraction of the textured structure. Another outlier exists (highlighted with the dotted violet circle) corresponding to another BCZT composition with a high FOM but with the lowest Φ.^[^ ^133^ ^]^ The low energy budget is attributed to the sol‐gel method used for the fabrication of the BCZT powder which has drastically decreased the calcination temperature (from >1000° to ≈700 °C). The optimization of the poling conditions and the sintering temperatures has also contributed to the obtained high FOM.

Unfortunately, still, all the BT‐based piezoceramics are inferior to the PZT‐5H and PZT‐5A in terms of the *k* values as shown in Figure 6h. The maximum *k*_p_ (≈0.6) has been superior to that of the PZT‐8 but only comparable to that of the PZT‐4.^[^ ^133^ ^]^ The rest of the BT‐based compositions exhibit much lower *k*_p_ values in the range of 0.3–0.55, possibly implying a substantial heating of the materials during the operation as an energy harvester, which may not be desirable in the deployment stage at the device level.

**Figure 7:**
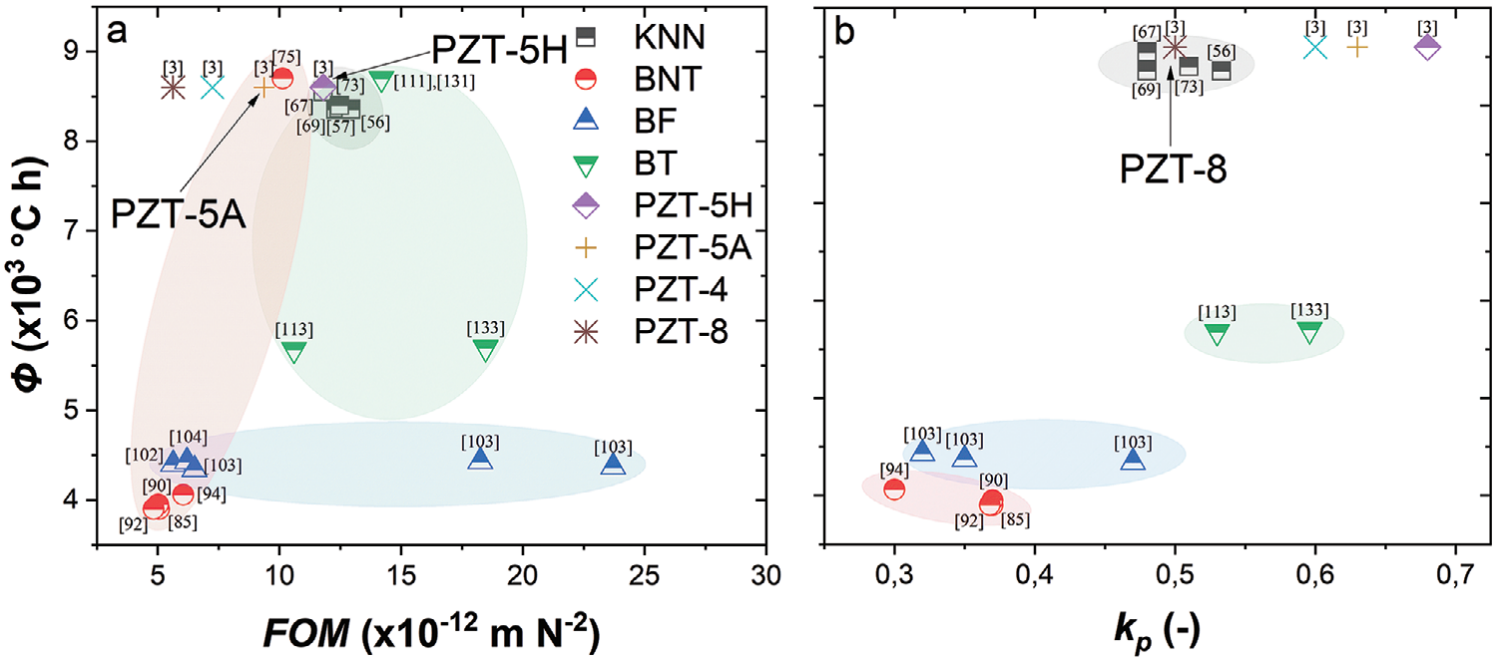
Comparison of the energy budget for fabrication with a) piezoelectric energy harvesting figure of merit and b) electromechanical coupling factor for different Pb‐based and Pb‐free piezoceramics.^[^ ^3^, ^56^, ^57^, ^67^, ^69^, ^73^, ^75^, ^85^, ^90^, ^92^, ^94^, ^102^, ^103^, ^104^, ^111^, ^113^, ^131^, ^133^ ^]^

As a summary, **Figure** **7** compares the most favorable or best‐performing Pb‐based and Pb‐free piezoceramics across different compositional families in terms of the FOM and *k*_p_ as a function of Φ. The figure indicates that some KNN‐based and the BCZT piezoceramics can be beneficial alternatives to the PZT family in solving the energy and environmental issues. Section 6 will provide detailed perspectives in this aspect.

## 4 Manufacturing Stage—Low‐Temperature Fabrication of Piezoceramics

For centuries, sintering has been a critical stage in the fabrication of advanced ceramics to reach the desired properties and there is no exception for piezoelectric ceramics, as has been discussed in Section 3.^[^ ^144^ ^]^ The appropriate sintering temperatures are usually >1000°C and hence a large part of the energy consumption in the production of the ceramics industry comes from the sintering stage, costing about 30% of the electricity used in the entire manufacturing process.^[^ ^145^ ^]^ In addition, high sintering temperatures lead to many drawbacks including not only elevated level of emissions but also severe unpredictable shrinkage of the materials that cause unwanted chemical diffusion and reaction between components. This makes piezoceramics incompatible with devices made from semiconductors, metals and polymers in the integration process which hinders the development of advanced multi‐component structures.^[^ ^146^, ^147^ ^]^ There is clearly a need to develop advanced sintering technologies that require lower temperatures to fabricate piezoceramics.

However, the high sintering temperatures have been very beneficial in terms of the piezoelectric properties obtained and therefore the biggest challenge is the retention of the original properties while attempting to reduce the temperature.^[^ ^148^ ^]^ This section will revisit some representative approaches that give hope of solving the challenge.

### 4.1 Reduction of Solid‐State Sintering Temperature with Sintering Aids

To make piezoelectric ceramics, different compounds as the reactants are mixed according to the target stoichiometry. For instance, PbO, TiO_2,_ and ZrO_2_ are mixed for the fabrication of PZT. The mixture of reactants will experience chemical reactions, which are known as the calcination stage as previously described. After calcination, powders containing ceramic particles are obtained and then the sintering process follows. Proper types and amounts of sintering aids such as the oxides of Ta, Bi, Li, Cu, and Sb can be added and mixed with the ceramic particles.^[^ ^149^ ^]^ The sintering aids decrease the sintering temperature by infiltrating into the matrix lattice and then promoting the formation of solid solutions. An appropriate selection of sintering aids can improve the densification and piezoelectric properties to some extent. Nevertheless, the achievement of reducing the sintering temperature remains limited to only 100–200 °C below the normally required temperatures. More aggressive approaches have also been developed.

### 4.2 Cold Sintering

Cold sintering takes advantage of the liquid phase sintering mechanism of ceramic sintering, wherein the solid ceramic particles coincide with a wetting liquid which has a lower melting temperature. The liquid flows between the solid particles to facilitate particle rearrangement and diffusion. After precipitation from the solution, ceramic particle densification is achieved at a lower temperature than that for the conventionally solid‐state sintered counterpart.^[^ ^150^, ^151^ ^]^ To compensate for the lost energy applied to the materials due to the reduced temperature, high pressure becomes an innate feature of cold sintering, which may result in a 50% energy saving.^[^ ^152^ ^]^ Typically, in the cold sintering process, ceramic particles densify in the presence of a transient liquid phase at temperatures below 350 °C while applying high pressures.^[^ ^153^ ^]^

In most cases, the concentration of the liquid phase varies between 1 and 10 vol% in the ceramic‐transient phase mixture.^[^ ^147^ ^]^ The driving force for the consolidation is contributed simultaneously by the liquid phase, the external pressure, and the mildly elevated temperature. The process shares an analogy to a combination of hot pressing and liquid‐assisted sintering.^[^ ^144^ ^]^ During the cold sintering process, a capillary force is formed under the applied uniaxial pressure, and the ratio between the concentration of the transient liquid and the corresponding pressure affects the particle rearrangement, dissolution, and precipitation and hence, the possible grain growth and densification.^[^ ^147^ ^]^

The detailed cold sintering process can be understood in two stages. First, the mixture of the ceramic powder and the liquid phase is compacted under the uniaxial pressure. The role of the liquid in this step is to lubricate the particles and consequently to stimulate sliding between particles as well as aiding the removal of the pressed materials from the die. Meanwhile, the sharp edges of the ceramic particles may dissolve in the liquid phase under the concentrated pressure, resulting in a better compaction compared to that of a dry environment.^[^ ^154^ ^]^ Secondly, while the ceramic is under pressure, the temperature is moderately increased. The solubility of the ceramic particles in the liquid phase subsequently increases, forming a supersaturated solution. As the supersaturated solution tends to evaporate at the elevated temperature, the mobility of the particles dissolved in the liquid phase increases. Eventually, each particle will be surrounded by the saturated solution owing to such a dissolution‐precipitation movement.^[^ ^155^ ^]^ When the elevated temperature reaches 120–350 °C, the above hydrated powders stream around each other to achieve a highly densified green body whilst the pressure is still active. Simultaneously, the transient liquid is either expelled out of the material or settles in the pores and/or at the grain boundaries, after which a densified ceramic is obtained at these low temperatures.

### 4.3 Low Temperature and Ultralow Temperature Co‐Fired Ceramics

The low temperature co‐fired ceramics (LTCC) and ultralow temperature co‐fired ceramics (ULTCC) are defined in comparison to their counterpart, the high temperature co‐fired ceramics (HTCC), based on the temperature ranges of fabrication utilized. The HTCC usually needs a processing temperature of >950°C while the LTCC and ULTCC need ranges of 700–950 °C and <700 °C, respectively.^[^ ^156^, ^157^, ^158^, ^159^ ^]^ Furthermore, the ULTCC can be classified into two categories: i) processed at 400–700 °C and ii) densified at as low as room temperature.^[^ ^157^ ^]^ The LTCC and ULTCC technologies have initially been developed as alternatives to printed circuit boards and thick film technologies but have then been used in the production of multilayer ceramic components, including piezoelectrics.^[^ ^160^, ^161^ ^]^

In these methods, additives of low melting point ceramic compounds and/or glasses are mixed with the matrix. The additives form liquid phases during sintering thus promoting grain growth similar to the cold sintering mechanism but without the high‐levels of pressure.^[^ ^162^, ^163^ ^]^ The reduced sintering temperature allows the ceramics to be co‐sintered with cost‐effective metals as the electrodes that have lower melting points compared to conventional solid‐state sintering temperatures, such as Ag and Cu.^[^ ^164^ ^]^ Using these additives, LTCC and ULTCC offer a large flexibility to the shaping of the ceramics. Slurries can be made with organic media and a wide range of shaping methods can be chosen and utilized, including tape casting, screen printing, and extrusion.^[^ ^165^ ^]^

Multilayer piezoelectric ceramics made with LTCC and ULTCC technologies not only contribute to potentially reduced energy consumption but also to ecofriendly operation in the deployment stage. For instance, micromotors based on multilayer piezoelectric actuators benefit from their shorter stabilization time, lower driving voltage, easier miniaturization and integration, and higher reliability compared to their monolayer counterparts.^[^ ^166^ ^]^

### 4.4 Room‐Temperature Densification

The concept of upside‐down composites has recently been proposed to fabricate piezoceramics at very close to room temperature. In this method, PZT ceramic particles are coated with Li_2_MoO_4_ (LMO) using water as the solvent wherein LMO is soluble in water. Here, the PZT and LMO act as the filler and the matrix as well as binder, respectively, similar to those of a conventional composite. However, the PZT particles are able to occupy much larger volumes than the LMO (matrix), which is opposite to that of a conventional composite, hence the term upside‐down composite has been introduced.^[^ ^167^ ^]^ The filler–matrix mixture is then hot‐pressed at only 120 °C to speed up the drying/evaporation process. The process can also be utilized to fabricate composites at room temperature provided that a prolonged drying time is used. The PZT‐LMO ceramic‐ceramic composite has exhibited *d*_33_ and *g*_33_ values of 84 pC N^−1^ and 33 mVm N^−1^, respectively.

Optimization of the selection and processing of the matrix helps to further improve the piezoelectric functionality. For instance, organotitanate (TiO*_x_*) has been used as the binder phase instead of LMO to improve the durability and electromechanical coupling of the composite because the organotitanate is not soluble in water and hence will not be influenced by the ambient humidity of the atmosphere. Here, the sol‐gel method has been used to produce the TiO*_x_* from a mixture of titanium isopropoxide as the precursor and 2‐methoxyethanol and ethanolamine as the solvent.^[^ ^146^ ^]^ Using the volume ratio of PZT:TiO*_x_* = 84:16, the composite has been fabricated by hot‐pressing under 250 MPa at 200–350 °C. The corresponding *d*_33_ and *g*_33_ values have increased to 150 pC N^−1^ and 53 mVm N^−1^, respectively.

**Table** **3** compares some representative piezoelectric ceramics made via the methods of the addition of sintering aid, the cold sintering, the LTCC and ULTCC and the room‐temperature densification, to their conventionally sintered counterparts. The apparent maximum processing temperature of the cold sintering method seems to be already sufficiently low (<350 °C). Meanwhile, the induced *d*, *g*, and FOM values at such a low temperature have been favorably retained at a level comparable to those of the corresponding high‐temperature sintered counterparts. However, these satisfactory functional properties are in fact not resultant from the low‐temperature process itself but instead are from a post‐annealing process at a substantially elevated temperature following the fabrication in the range of 700–900 °C.

**Table 3:** Summary of representative piezoelectric ceramics made by the low‐temperature and ordinary‐temperature sintering methods

Material	Sintering method	Maximum processing temperature [°C]	*d*_33_ [pC N^−1^]	*g*_33_ [mVm N^−1^]	FOM [x10^−12^ m^2^ N^−1^]	Refs.
(K_0.5_Na_0.5_)NbO_3_	Conventional solid‐state sintering	1020–1130	≈110	≈21	≈2.2	[168]
(K_0.5_Na_0.5_)NbO_3_	Cold sintering with water as the transient liquid	120	≈130	≈1.4	≈0.2	[169]
(K_0.5_Na_0.5_)NbO_3_	Cold sintering with KOH and NaOH solutions as the transient phase	350	≈150	≈20	≈3	[170]
Mn‐doped (K_0.5_Na_0.5_)NbO_3_	Cold sintering with KMnO_4_ aqueous solution as the transient phase	120	≈145	≈20	≈2.9	[171]
(Na_0.5_Bi_0.5_)TiO_3_	Conventional solid‐state sintering	1150‐1200	≈60	≈15	≈0.9	[172]
(Na_0.5_Bi_0.5_)TiO_3_	Cold sintering with Bi(NO_3_)_3_/NaOH/TiO_2_ suspension as the transient phase	180	≈50	≈9	≈0.5	[173]
Soft‐PZT	Conventional solid‐state sintering	1250	≈590	≈20	≈11.7	[174]
PZT	Cold sintering with Pb(NO_3_)_2_ as the transient phase	300	≈200	≈17	≈3.3	[175]
PZT	Room‐temperature densification with Li2MoO4 as the matrix/binder	120	≈85	≈30	≈2.5	[167]
PZT	Room‐temperature densification with TiO*_x_* as the matrix/binder	350	≈150	≈50	≈7.8	[146]
PNN‐PMW‐PZT	LTCC with MnO_2_ as the sintering aid	1000	≈345	≈38	≈13.1	[165]
Pb(Ni_1/3_Nb_2/3_)‐PZT	Li_2_CO_3_ as the sintering aid	950	≈690	≈18	≈12.6	[176]

In comparison, the upside‐down composites made with the room‐temperature densification method seem to exhibit significantly low *d* and FOM values due to the low processing temperature where post‐annealing at an elevated temperature is absent. However, the *g* values measured from the upside‐down composites are always higher than those of corresponding conventionally sintered ceramics owing to the reduced effective permittivity. Such a phenomenon appears when a high‐permittivity filler (e.g., PZT particles) and a low‐permittivity binder (e.g., LMO or TiO*_x_*) coexist in a 0–3 composite (filler particles randomly dispersed in the binder matrix). A greater difference between the permittivity values of the filler and binder results in more dominance of the binder over the permittivity, due to the lack of interconnectivity between the fillers which leads to a concentration of the external electric field on the low‐permittivity binder rather than on the filler during the dielectric measurement.^[^ ^177^, ^178^, ^179^, ^180^ ^]^

It is worth pointing out that the upside‐down composite method uses crushed sintered ceramics as the filler particles. This, on the one hand, may not be favorable for the actual energy saving in practice because the ceramics must be sintered through the conventional, high‐temperature route in the first place. This issue needs to be solved before this method can make a true impact on the low‐temperature fabrication of high‐performance piezoceramics for the purpose of energy saving. Research directions contributing toward solving the issue will be discussed in Section 6. On the other hand, piezoceramic components made via the conventional sintering procedure available after their retirement or breakdown become ideal material sources for the upside–down composites. This may offer an immediate benefit for recycling and reusing old piezoceramics. The following section discusses this aspect.

## 5 Recycling of Piezoceramics

With the constantly increasing environmental concerns, novel solutions for electronic waste handling, recycling, and reusing are urgently needed. In 2019, the amount of electronic waste was calculated to be nearly 54 million tons, of which only roughly 17% was properly recycled while the majority ended up in landfills.^[^ ^181^ ^]^ Among all the electronic wastes, those of small IT devices such as laptops and mobile phones counted for 4.7 million tons, which indicates a huge potential for recycling raw materials. Particularly, electroceramic waste is estimated to be about 700 thousand tons where the piezoelectric devices take a share of about 17%.^[^ ^181^, ^182^, ^183^, ^184^ ^]^ It can then be concluded that there are tens of thousands of tons of piezoelectric waste available worldwide.

In addition to used components, the manufacturing of electroceramics commonly leave rejection of certain products due to, e.g., material deformation, cracking, and uneven densification during the sintering process. These leftover products are usually disposed of without being properly used in any scenario.

The upside‐down composites introduced above in Section 4 contain an exceptionally high content of filler materials in multimodal particle sizes with the majority being around 100 µm.^[^ ^146^, ^167^, ^185^ ^]^ Obviously, the most convenient and viable way to achieve such large particles is to retrieve the wasted (retired or rejected) ceramics and then crush them into the desired particle sizes. The authors, in their recent research works, have proved that the upside‐down composite method is transferrable to different Pb‐based and Pb‐free piezoceramics.^[^ ^180^, ^186^ ^]^ When a suitable filler‐binder combination is selected, such as the oxide‐halide perovskites, the recycling of piezoceramics can even be achieved simultaneously with the recycling of perovskite solar cells. This spans the recycling from kinetic energy harvesters to light energy harvesters in which the Pb‐containing perovskites are also under issue for future high‐performance solar cells.^[^ ^186^, ^187^ ^]^

Therefore, it is believed that the upside‐down composite method is a promising future enabler for recycling and reuse of energy harvesters that will have a broad impact where the high‐performance harvesters made from the energy‐intensive approaches can be converted to slightly worse performing but still feasible ones for less demanding applications at no significant excessive cost of energy or environmental compromise.

## 6 Summary and Perspectives

This paper has discussed the issues faced by the piezoelectrics research and industry communities with respect to energy and environmental issues. The paper has revisited the aspects of four stages in the materials’ lifecycle: i) energy saving from reliable material prediction and design, ii) energy saving in manufacturing, iii) trade‐offs between the energy and environmental benefits considering the deployment in the energy harvesting technology, and iv) recycling and reuse of waste materials.

### 6.1 Materials Design and Calculation

The theoretical studies in the field of ferroelectric oxide perovskites that possess the potential to offer excellent piezoelectric properties have made impressive progress and effort in rationalizing, understanding, and explaining the rich behavior of Pb‐based perovskites. It has been shown that several key properties of the ferroelectric oxide perovskite solid solutions can be predicted, which suggests that these complex material properties are governed by simple physical relationships. The successful attempts, especially in understanding the PbTiO_3_‐based solid solutions, set the milestone before starting to understand the environmentally friendly Pb‐free ferroelectrics.

Due to their excellent properties, Pb‐based solid solutions still dominate the market. The hazardous nature of Pb urgently demands substitutes but necessary theoretical support for predicting excellent Pb‐free perovskites in order to propel them in this direction is lacking. The development of Pb‐free ferroelectric oxide perovskites is hindered by their smaller energy scale and lower ferroelectric response. In addition, the Pb‐free candidates usually show a higher complexity level compared to the PbTiO_3_‐based materials. Therefore, future theories and models must be developed towards being able to reliably handle more complex structure–property correlations. A promising approach is the multilevel modeling based on machine learning using similar logics as applied in the Pb‐based counterparts.

It is known that minor elemental doping in ferroelectric oxide perovskites plays a significant role in enhancing piezoelectric properties. The factor of doping also needs to be addressed in future theoretical models. Being an extrinsic phenomenon, doping degrades the reliability of modeling due to a variety of local and non‐local compositional and structural variations.

Another important aspect that requires attention is the responsive properties of these complex perovskite oxide solid solutions. Many factors could play an important role in piezoelectric properties. For piezoelectric ceramics, the properties are strongly affected by microstructure and hence it is quintessential to shed light on the effects of composition and different cations on the microstructure. The current theoretical approaches to evaluate piezoelectric properties (e.g., *d*_33_) are cumbersome and time‐consuming. There is a need to build a new model at least for the reliable prediction of *d*_33_. This could be achieved by using the available data and previously known correlations whilst taking advantage of machine learning to build a hybrid model.

### 6.2 Balance between Energy and Environmental Benefits

Among currently available Pb‐free oxide perovskites that have been validated in experiments, the KNN‐based piezoceramics exhibit comparable energy harvesting capabilities and they consume a similar amount of energy in manufacture compared to the PZT‐5H which is considered an excellent Pb‐based piezoceramic for energy harvesting. The electromechanical coupling of the KNN‐based ceramics tends to be weaker than that of the PZT‐5H, making the KNN‐based ones liable to heat more during the resonant operation of piezoelectric energy harvesters and thus decrease their efficiency and cause a waste of energy. Therefore, the future of KNN‐based piezoceramics requires a significant increase in the electromechanical coupling factor. However, it is to be noted that the KNN‐based piezoceramics generally have a high *T*_c_ (>200 °C), and thanks to this, the heating caused by the weak electromechanical coupling might not be a big issue. More device‐level tests integrating the KNN‐based components should be done in the future to reach more conclusive decisions on the potential use of these materials in energy harvesters.

There are also some other issues remaining with the KNN‐based ceramics, including their poor temperature stability due to a temperature‐dependent phase boundary, and the hygroscopicity of the raw powders.^[^ ^4^, ^188^ ^]^ These issues should be addressed for energy harvesting applications as well in the future. Nevertheless, in terms of performance, the results represent a strong incentive to mass‐produce KNN‐based ceramics for piezoelectric energy harvesters.

The BNT‐based ceramics do not seem to present a good incentive to be used in energy harvesters due to their mediocre energy harvesting performance and electromechanical coupling. However, the energy budgets needed for their fabrication are much lower than those of the PZT family, giving some motivation for using BNT‐based ceramics in the scenario where energy saving during fabrication is a crucial factor. From this aspect, the BNT‐based ceramics would make an impact in the energy harvesting sector if higher piezoelectricity would be achieved in the future. It also should be noted that the BNT‐based ceramics suffer from issues such as low *T*_d_, which may introduce an additional barrier before they are adopted as promising Pb‐free compositions for piezoelectric energy harvesters.^[^ ^4^ ^]^

The BF‐based piezoceramics have a mixed energy harvesting performance with some performing significantly better than the PZT‐5H while others perform much worse. Meanwhile, all the BF‐based ceramics show much weaker electromechanical coupling than that of the PZT‐5H, despite their energy budgets of fabrication also being much lower. Like the BNT‐based ceramics, the BF‐based ceramics may also increase interest in their being used in the energy harvesting sector thanks to the smaller energy footprint of manufacturing if the inherently low *d*_33_ can be boosted in the future. Promising research routes include the addition of excessive bismuth and super‐tetragonal dopants, which in turn requires the development of theoretical models for the prediction of high‐performance BF‐based piezoelectrics to reduce the need for experimental trials and consequently speed up the pace development. The high *T*_c_ of the BF‐based ceramics may also attract the energy harvesting sector before the fabrication and processing difficulties are eased, which include a compulsory quenched cooling procedure after sintering to avoid the formation of impurity phases which makes the fabrication difficult, and the high coercive field which makes the poling difficult.

The BT‐based piezoceramics show the best match with the PZT‐5H in terms of the energy harvesting performance, with some compositions also requiring much lower energy budgets thanks to the optimized alternative fabrication routes which shed light on their possible usage for fabricating other Pb‐free compositions in addition to reducing their overall energy budget. Even though all the Pb‐free compositions exhibit weaker electromechanical coupling than the PZT‐5H, the BT‐based ceramics exhibit the highest level among all the Pb‐free members.

Overall, the BCZT‐based piezoceramics are considered the most sustainable and viable substitutes for PZT in the energy harvesting sector. In future research, the TGG method should be further developed in combination with different types of low‐temperature fabrication methods including the hydrothermal and sol‐gel routes for powder synthesis and calcination as well as the approaches of cold sintering and room‐temperature densification. These developments are expected to further improve the feasibility of the BCZT‐based ceramics in addressing energy and environmental challenges. The only drawback of these piezoceramics is the low *T*_c_ (≈90–100 °C). Considering the relatively weak electromechanical coupling, the potentially elevated level of heating during the operation of energy harvesters may impose an inevitable problem as the heating will destabilize and degrade the superior functionality. Therefore, the BCZT‐based ceramics may only be suitable for applications at close to room temperature with a cool ambient environment that requires a light working load.

Future research works may focus on the increase of the *T*_c_ and electromechanical coupling for the BCZT‐based ceramics. Advanced machine learning models are to be relied on in combination with experimental validation. It is admitted that this study has not considered the mechanical properties of the piezoceramics in question, although mechanical properties may also largely affect the performance of piezoelectric energy harvesters in the dynamic environment. However, the lack of evaluation of mechanical properties does not bias the conclusions reached in this article, i.e., the general suitability of the KNN‐based and the BCZT ceramics as energy‐ and environment‐beneficial candidates to replace the Pb‐based counterparts for energy harvesting applications. Potential future works on the evaluation of the mechanical properties for these two types of Pb‐free compositions will only enrich the information in terms of optimizing the device‐level performance, which is certainly a positive and useful addition rather than a potential contradiction to this study.

### 6.3 Energy Saving and Materials Recycling

Cold sintering and the room‐temperature densification methods are considered promising for the future fabrication of high‐performance piezoceramics which facilitate energy saving during the low‐temperature process that supplements or even replaces the conventional high‐temperature sintering procedure. The room‐temperature densification method holds advantages over the cold sintering approach because post‐annealing at the elevated temperature can be avoided for the upside‐down composites. The major issue is that the filler materials must be well‐sintered ceramic particles. Therefore, future research on the upside–down composites should address the challenges of making the source ceramic particles at low temperatures.

In this respect, the hydrothermal synthesis and sintering route looks interesting. Fabrication of piezoceramics through the hydrothermal route is less popular compared to the cold sintering, perhaps due to the long processing period and much lower material yield.^[^ ^147^, ^189^ ^]^ Nevertheless, this method shares many similarities with the widely understood hydrothermal chemistry, and more importantly, the calcination and sintering stages can be merged, which may promote more effective energy saving. In this method, either the raw powders or the ceramic powders are pressed mechanically in an autoclave at temperatures below 350°C and under relatively mild hydrothermal ambience.^[^ ^189^ ^]^ This method offers several unique advantages, including low reaction temperatures, and results in high‐quality crystals of high purity with controllable size and morphology, homogenous nucleation, and the prevention of unwanted off‐stoichiometry due to elemental volatility such as Pb, Bi, and alkali elements.^[^ ^190^, ^191^, ^192^ ^]^

It has been proven that hydrothermal sintering can successfully deposit piezoelectric thin films on 3D substrates.^[^ ^193^ ^]^ Future attempts should be made to increase the material yield. This low‐cost and less‐polluting method should certainly be used in combination with the upside‐down composites, which will eventually offer an eco‐friendly choice for low‐temperature fabrication of Pb‐free piezoceramics.

Last but not least, future research should also head towards recycling piezoceramics, and even simultaneous recycling of oxide perovskite piezoceramics and halide perovskite solar cells, using the upside‐down composite method to give a second life to waste materials and components. The remade materials may show slightly worse properties than their pristine form but will greatly reduce the energy and environmental cost when being used to perform less‐demanding functional tasks. For example, the upside‐down ceramic‐ceramic piezoelectric composites have already shown an even better sensing capability than pristine piezoceramics. This early success makes further efforts in this research direction considerably attractive.

## Conflict of Interest

The authors declare no conflict of interest.

## References

[1] F. Li , M. J. Cabral , B. Xu , Z. Cheng , E. C. Dickey , J. M. LeBeau , J. Wang , J. Luo , S. Taylor , W. Hackenberger , L. Bellaiche , Z. Xu , L.‐Q. Chen , T. R. Shrout , S. Zhang , Science 2019, 364, 264. 31000659 10.1126/science.aaw2781

[2] F. Li , D. Lin , Z. Chen , Z. Cheng , J. Wang , C. Li , Z. Xu , Q. Huang , X. Liao , L.‐Q. Chen , T. R. Shrout , S. Zhang , Nat. Mater. 2018, 17, 349. 29555999 10.1038/s41563-018-0034-4

[3] T. Shrout , S. Zhang , J. Electroceram. 2007, 19, 113.

[4] T. Zheng , J. Wu , D. Xiao , J. Zhu , Prog. Mater. Sci 2018, 98, 552.

[5] K. S. Ramadan , D. Sameoto , S. Evoy , Smart Mater. Struct. 2014, 23, 033001.

[6] S. Mishra , L. Unnikrishnan , S. K. Nayak , S. Mohanty , Macromol. Mater. Eng 2019, 304, 1800463.

[7] M. E. Lines , A. M. Glass , Principles and Applications of Ferroelectrics and Related Materials, Oxford University Press, Oxford 2001.

[8] H. F. Tiersten , G. A. Coquin , F. S. Welsh III , in IEEE Standard on Piezoelectricity, *ANSI/IEEE Std* (Eds: A. H. Meitzler , D. Berlincourt , F. S. Welsh III , H. F. Tiersten , G. A. Coquin , A. W. Warner ) IEEE, Piscataway, NJ 1988.

[9] Y. Bai , Ph.D. Thesis, University of Birmingham, xx 2015.

[10] Y. Bai , P. Tofel , J. Palosaari , H. Jantunen , J. Juuti , Adv. Mater 2017, 29, 1700767. 10.1002/adma.201700767 28585344

[11] IEA Digitalisation, IEA, Paris , 2022, https://www.iea.org/reports/digitalisation (accessed March 2023).

[12] B. Noheda , D. E. Cox , G. Shirane , J. A. Gonzalo , L. E. Cross , S.‐E. Park , Appl. Phys. Lett 1999, 74, 2059.

[13] B. Noheda , D. E. Cox , G. Shirane , S.‐E. Park , L. E. Cross , Z. Zhong , Phys. Rev. Lett 2001, 86, 3891. 11329350 10.1103/PhysRevLett.86.3891

[14] Z.‐G. Ye , B. Noheda , M. Dong , D. Cox , G. Shirane , Phys. Rev. B 2001, 64, 184114.

[15] S. Yadav , I. Grinberg , J. Appl. Phys 2021, 129, 174101.

[16] L. Bellaiche , A. García , D. Vanderbilt , Phys. Rev. Lett 2000, 84, 5427. 10990960 10.1103/PhysRevLett.84.5427

[17] I. Grinberg , Y.‐H. Shin , A. M. Rappe , Phys. Rev. Lett 2009, 103, 197601. 20365954 10.1103/PhysRevLett.103.197601

[18] L.‐Q. Chen , J. Am. Ceram. Soc. 2008, 91, 1835.

[19] J.‐J. Wang , B. Wang , L.‐Q. Chen , Annu. Rev. Mater. Res. 2019, 49, 127.

[20] D. E. Cox , B. Noheda , G. Shirane , Y. Uesu , K. Fujishiro , Y. Yamada , Appl. Phys. Lett 2001, 79, 400.

[21] T. Shiosaki , Ferroelectrics 1989, 91, 39.

[22] S. Zhang , T. R. Shrout , IEEE Trans. Ultrason. Ferroelectr. Freq. Control 2010, 57, 2138. 20889397 10.1109/TUFFC.2010.1670 PMC3180875

[23] J. Tian , P. Han , X. Huang , H. Pan , J. F. Carroll , D. A. Payne , Appl. Phys. Lett 2007, 91, 222903.

[24] G. Burns , F. H. Dacol , Solid State Commun. 1983, 48, 853.

[25] M. Eremenko , V. Krayzman , A. Bosak , H. Playford , K. Chapman , J. Woicik , B. Ravel , I. Levin , Nat. Commun. 2019, 10, 2728. 31227698 10.1038/s41467-019-10665-4 PMC6588601

[26] D. Fu , H. Taniguchi , M. Itoh , S. Koshihara , N. Yamamoto , S. Mori , Phys. Rev. Lett. 2009, 103, 207601. 20366012 10.1103/PhysRevLett.103.207601

[27] S. Tsukada , K. Ohwada , H. Ohwa , S. Mori , S. Kojima , N. Yasuda , H. Terauchi , Y. Akishige , Sci. Rep. 2017, 7, 17508. 29235499 10.1038/s41598-017-17349-3 PMC5727483

[28] H. Takenaka , I. Grinberg , S. Liu , A. M. Rappe , Nature 2017, 546, 391. 28617453 10.1038/nature22068

[29] V. A. Isupov , Crystallogr. Rep. 2004, 49, 719.

[30] I. W. Chen , J. Phys. Chem. Solids 2000, 61, 197.

[31] A. Samanta , S. Yadav , Z. Gu , C. J. G. Meyers , L. Wu , D. Chen , S. Pandya , R. A. York , L. W. Martin , J. E. Spanier , I. Grinberg , Adv. Mater. 2022, 34, 2106021. 10.1002/adma.202106021 34695263

[32] I. Grinberg , M. R. Suchomel , P. K. Davies , A. M. Rappe , J. Appl. Phys 2005, 98, 094111.

[33] W. Ma , L. E. Cross , Appl. Phys. Lett. 2002, 81, 3440.

[34] P. Zubko , G. Catalan , A. K. Tagantsev , Annu. Rev. Mater. Res. 2013, 43, 387.

[35] D.‐S. Park , M. Hadad , L. M. Riemer , R. Ignatans , D. Spirito , V. Esposito , V. Tileli , N. Gauquelin , D. Chezganov , D. Jannis , J. Verbeeck , S. Gorfman , N. Pryds , P. Muralt , D. Damjanovic , Science 2022, 375, 653. 35143321 10.1126/science.abm7497

[36] S. C. Abrahams , S. K. Kurtz , P. B. Jamieson , Phys. Rev. 1968, 172, 551.

[37] I. Grinberg , A. M. Rappe , Phys. Rev. B 2004, 70, 220101.

[38] I. Grinberg , A. M. Rappe , Phys. Rev. Lett. 2007, 98, 37603. 10.1103/PhysRevLett.98.037603 17358730

[39] S. Yadav , A. Samanta , O. Shafir , Adv. Mater. 2022, 34, 2106105. 10.1002/adma.202106105 34811814

[40] W. J. Merz , J. Appl. Phys. 1956, 27, 938.

[41] B. Meyer , D. Vanderbilt , Phys. Rev. B 2002, 65, 104111.

[42] T. Tybell , P. Paruch , T. Giamarchi , J.‐M. Triscone , Phys. Rev. Lett. 2002, 89, 97601. 10.1103/PhysRevLett.89.097601 12190438

[43] Y.‐H. Shin , I. Grinberg , I.‐W. Chen , A. M. Rappe , Nature 2007, 449, 881. 17922002 10.1038/nature06165

[44] S. Liu , I. Grinberg , A. M. Rappe , Nature 2016, 534, 360. 27306186 10.1038/nature18286

[45] I. Grinberg , Ferroelectrics: Advances in Fundamental Studies and Emerging Applicaitons (Eds: Y. Bai , I. Grinberg ), IOP Publishing, 2022.

[46] P. V Balachandran , B. Kowalski , A. Sehirlioglu , T. Lookman , Nat. Commun 2018, 9, 1668. 29700297 10.1038/s41467-018-03821-9 PMC5920103

[47] V. R. Cooper , K. M. Rabe , Phys. Rev. B 2009, 79, 180101.

[48] S. V Glushanin , V. Y. Topolov , A. V. Turik , Crystallogr. Rep. 2003, 48, 491.

[49] A. Harzellaoui , O. Arbouche , K. Amara , J. Comput. Electron. 2020, 19, 1365.

[50] Directive 2002/95/EC of the European parliament and of the council of 27 January 2003 on the restriction of the use of certain hazardous substances in electrical and electronic equipment, Official Journal of the European Union, L37/19‐23 2003, https://eur‐lex.europa.eu/legal‐content/EN/TXT/PDF/?uri=CELEX:32002L0095&from=EN (accessed: March 2023).

[51] Study to assess requests for a renewal of nigh exemptions of Annex III of Directive 2011/65/EU – Final Report, https://rohs.exemptions.oeko.info/fileadmin/user_upload/RoHS_Pack_22/RoHS_Pack‐22_final_report_amended_February_2022.pdf( accessed: March 2023).

[52] Directive 2002/95/EC of the European parliament and of the council of 27 January 2003 on the restriction of the use of certain hazardous substances in electrical and electronic equipment, 2002L0095, https://eur‐lex.europa.eu/LexUriServ/LexUriServ.do?uri=CONSLEG:2002L0095:20110910:EN:PDF (Accessed: March 2023).

[53] J. Rödel , J.‐F. Li , MRS Bull. 2018, 43, 576.

[54] A. J. Bell , O. Deubzer , MRS Bull. 2018, 43, 581.

[55] W. V Beveren , Y. Saito , H. Takao , T. Tani , T. Nonoyama , K. Takatori , T. Homma , T. Nagaya , M. Nakamura , Nature 2004, 432, 84. 15516921 10.1038/nature03028

[56] B. Wu , H. Wu , J. Wu , D. Xiao , J. Zhu , S. J. Pennycook , J. Am. Chem. Soc. 2016, 138, 15459. 27933925 10.1021/jacs.6b09024

[57] T. Zheng , H. Wu , Y. Yuan , X. Lv , Q. Li , T. Men , C. Zhao , D. Xiao , J. Wu , K. Wang , J.‐F. Li , Y. Gu , J. Zhu , S. J. Pennycook , Energy Environ. Sci. 2017, 10, 528.

[58] J. G. Wu , D. Q. Xiao , J. G. Zhu , Chem. Rev. 2015, 115, 2559. 25792114 10.1021/cr5006809

[59] R. Zuo , C. Ye , X. Fang , J. Phys. Chem. Solids 2008, 69, 230.

[60] B. Zhang , J. Wu , X. Wang , X. Cheng , J. Zhu , D. Xiao , Curr. Appl. Phys. 2013, 13, 1647.

[61] R. Zuo , C. Ye , X. Fang , Jpn. J. Appl. Phys. 2007, 46, 6733.

[62] J. Wu , H. Tao , Y. Yuan , X. Lv , X. Wang , X. Lou , RSC Adv. 2015, 5, 14575.

[63] R. Zuo , J. Fu , D. Lv , Y. Liu , J. Am. Ceram. Soc. 2010, 93, 2783.

[64] Y. Gao , J. Zhang , Y. Qing , Y. Tan , Z. Zhang , X. Hao , J. Am. Ceram. Soc. 2011, 94, 2968.

[65] J. Fu , R. Zuo , X. Wang , L. Li , J. Phys. D: Appl. Phys. 2009, 42, 012006.

[66] R. Zuo , J. Fu , D. Lv , J. Am. Ceram. Soc 2009, 92, 283.

[67] X. P. Wang , J. G. Wu , D. Q. Xiao , J. G. Zhu , X. J. Cheng , T. Zheng , B. Y. Zhang , X. J. Lou , X. J. Wang , J. Am. Chem. Soc. 2014, 136, 2905. 24499419 10.1021/ja500076h

[68] X. Wang , J. Wu , D. Xiao , X. Cheng , T. Zheng , X. Lou , B. Zhang , J. Zhu , ACS Appl. Mater. Interfaces 2014, 6, 6177. 24784228 10.1021/am500819v

[69] T. Zheng , J. Wu , D. Xiao , J. Zhu , X. Wang , X. Lou , J. Mater. Chem. A 2015, 3, 1868.

[70] K. Xu , J. Li , X. Lv , J. G. Wu , X. X. Zhang , D. Q. Xiao , J. G. Zhu , Adv. Mater. 2016, 28, 8519. 27441456 10.1002/adma.201601859

[71] X. Lv , J. Wu , D. Xiao , H. Tao , Y. Yuan , J. Zhu , X. Wang , X. Lou , Dalton Trans. 2015, 44, 4440. 25679361 10.1039/c4dt04038d

[72] B. Wu , J. Wu , D. Xiao , J. Zhu , Dalton Trans. 2015, 44, 21141. 26598931 10.1039/c5dt03680a

[73] X. Lv , Z. Li , J. Wu , J. Xi , M. Gong , D. Xiao , J. Zhu , Mater. Des. 2016, 109, 609.

[74] X. Lv , J. Wu , S. Yang , D. Xiao , J. Zhu , ACS Appl. Mater. Interfaces 2016, 8, 18943. 27404481 10.1021/acsami.6b04288

[75] B. Parija , T. Badapanda , S. Panigrahi , T. P. Sinha , J. Mater. Sci. Mater. Electron. 2013, 24, 402.

[76] C. Xu , D. Lin , K. W. Kwok , Solid State Sci. 2008, 10, 934.

[77] B.‐H. Kim , S.‐J. Han , J.‐H. Kim , J.‐H. Lee , B.‐K. Ahn , Q. xu , Ceram. Int. 2007, 33, 447.

[78] J. Anthoniappen , C.‐H. Lin , C. S. Tu , P.‐Y. Chen , C.‐S. Chen , S.‐J. Chiu , H.‐Y. Lee , S.‐F. Wang , J. Am. Ceram. Soc. 2014, 97, 1890.

[79] W.‐C. Lee , C.‐Y. Huang , L.‐K. Tsao , Y.‐C. Wu , J. Eur. Ceram. Soc. 2009, 29, 1443.

[80] B. Wu , D. Xiao , W. Wu , J. Zhu , Q. Chen , J. Wu , Ceram. Int. 2012, 38, 5677.

[81] K. Yoshii , Y. Hiruma , H. Nagata , T. Takenaka , Jpn. J. Appl. Phys. 2006, 45, 4493.

[82] Y.‐R. Zhang , J.‐F. Li , B.‐P. Zhang , J. Am. Ceram. Soc. 2008, 91, 2716.

[83] A. Moosavi , M. A. Bahrevar , A. R. Aghaei , P. Ramos , M. Algueró , H. Amorín , J. Phys. D: Appl. Phys. 2014, 47, 055304.

[84] L. Yunwen , D. Xiao , D. Lin , J. Zhu , L. Wu , X. Wang , Ceram. Int. 2007, 33, 1445.

[85] D. Lin , D. Xiao , J. Zhu , P. Yu , Appl. Phys. Lett. 2006, 88, 062901.

[86] Y. Hiruma , H. Nagata , T. Takenaka , J. Appl. Phys. 2008, 104, 124106.

[87] A. Singh , R. Chatterjee , J. Am. Ceram. Soc. 2013, 96, 509.

[88] F. Weyland , M. Acosta , J. Koruza , P. Breckner , J. Rödel , N. Novak , Adv. Funct. Mater. 2016, 26, 7326.

[89] Y. Hiruma , H. Nagata , T. Takenaka , Ceram. Int. 2009, 35, 117.

[90] D. Lin , Q. Zheng , C. Xu , K. W. Kwok , Appl. Phys. A 2008, 93, 549.

[91] B. Wu , C. Han , D. Xiao , Z. Wang , J. Zhu , J. Wu , Mater. Res. Bull. 2012, 47, 3937.

[92] D. Lin , K. W. Kwok , H. L. W. Chan , Solid State Ionics 2008, 178, 1930.

[93] G. Fan , W. Lu , X. Wang , F. Liang , Appl. Phys. Lett. 2007, 91, 202908.

[94] Y.‐J. Dai , S. Zhang , T. R. Shrout , X.‐W. Zhang , J. Am. Ceram. Soc. 2010, 93, 1108.

[95] X. Chen , J. Wang , G. Yuan , D. Wu , J. Liu , J. Yin , Z. Liu , J. Alloys Compd. 2012, 541, 173.

[96] Y. Yao , W. Liu , Y. Chan , C. Leung , C. Mak , B. Ploss , Int. J. Appl. Ceram. Technol. 2011, 8, 1246.

[97] T. Zheng , J. Wu , J. Mater. Chem. C 2015, 3, 3684.

[98] Z. Cen , C. Zhou , H. Yang , Q. Zhou , W. Li , C. Yan , L. Cao , J. Song , L. Peng , J. Am. Ceram. Soc 2013, 96, 2252.

[99] Q. Zhou , C. Zhou , H. Yang , C. Yuan , G. Chen , L. Cao , Q. Fan , J. Mater. Sci. Mater. Electron. 2014, 25, 196.

[100] C. Zhou , Z. Cen , H. Yang , Q. Zhou , W. Li , C. Yuan , H. Wang , Phys. B 2013, 410, 13.

[101] H. Yang , C. Zhou , X. Liu , Q. Zhou , G. Chen , W. Li , H. Wang , J. Eur. Ceram. Soc. 2013, 33, 1177.

[102] T. Zheng , Y. Ding , J. Wu , RSC Adv. 2016, 6, 90831.

[103] M. H. Lee , D. J. Kim , J. S. Park , S. W. Kim , T. K. Song , M.‐H. Kim , W.‐J. Kim , D. Do , Adv. Mater. 2015, 27, 6976. 26444562 10.1002/adma.201502424

[104] T. Zheng , Z. Jiang , J. Wu , Dalton Trans. 2016, 45, 11277. 27357104 10.1039/c6dt01805j

[105] L. Luo , N. Jiang , F. Lei , Y. Guo , Q. Zheng , D. Lin , J. Mater. Sci. Mater. Electron. 2014, 25, 1736.

[106] D. Lin , Q. Zheng , Y. Li , Y. Wan , Q. Li , W. Zhou , J. Eur. Ceram. Soc. 2013, 33, 3023.

[107] Y. Li , Y. Guo , Q. Zheng , K. H. Lam , W. Zhou , Y. Wan , D. Lin , Mater. Res. Bull. 2015, 68, 92.

[108] C. Zhou , A. Feteira , X. Shan , H. Yang , Q. Zhou , J. Cheng , W. Li , H. Wang , Appl. Phys. Lett. 2012, 101, 032901.

[109] Q. Zhou , C. Zhou , H. Yang , G. Chen , W. Li , H. Wang , J. Am. Ceram. Soc. 2012, 95, 3889.

[110] M. M. Vijatović Petrović , J. D. Bobić , B. Stojanović , Sci. Sintering 2008, 40, 235.

[111] W. Liu , X. Ren , Phys. Rev. Lett. 2009, 103, 257602. 20366285 10.1103/PhysRevLett.103.257602

[112] P.‐F. Zhou , B.‐P. Zhang , L. Zhao , X.‐K. Zhao , L.‐F. Zhu , L.‐Q. Cheng , J.‐F. Li , Appl. Phys. Lett. 2013, 103, 172904.

[113] P. Wang , Y. Li , Y. Lu , J. Eur. Ceram. Soc. 2011, 31, 2005.

[114] E. Chandrakala , J. Paul Praveen , A. Kumar , A. R. James , D. Das , J. Am. Ceram. Soc. 2016, 99, 3659.

[115] Y. Liu , Y. Chang , F. Li , B. Yang , Y. Sun , J. Wu , S. Zhang , R. Wang , W. Cao , ACS Appl. Mater. Interfaces 2017, 9, 29863. 28799748 10.1021/acsami.7b08160

[116] L.‐F. Zhu , B.‐P. Zhang , X.‐K. Zhao , L. Zhao , P.‐F. Zhou , J.‐F. Li , J. Am. Ceram. Soc. 2013, 96, 241.

[117] L.‐F. Zhu , B.‐P. Zhang , L. Zhao , J.‐F. Li , J. Mater. Chem. C 2014, 2, 4764.

[118] L. Zhao , B.‐P. Zhang , Z. Zhou , P. Zhu , L.‐F. , N. Wang , Ceram. Int. 2016, 42, 1086.

[119] X. Wang , X. Chao , P. Liang , L. Wei , Z. Yang , Ceram. Int. 2014, 40, 9389.

[120] C. zhao , H. Wang , J. Xiong , J. Wu , Dalton Trans. 2016, 45, 6466. 26952807 10.1039/c5dt04891e

[121] L.‐F. Zhu , B.‐P. Zhang , L. Zhao , S. Li , Y. Zhou , X.‐C. Shi , N. Wang , J. Eur. Ceram. Soc. 2016, 36, 1017.

[122] L.‐F. Zhu , B.‐P. Zhang , X.‐K. Zhao , L. Zhao , F.‐Z. Yao , X. Han , P.‐F. Zhou , J.‐F. Li , Appl. Phys. Lett 2013, 103, 072905.

[123] Y. Cui , X. Liu , M. Jiang , Y. Hu , Q. Su , H. Wang , J. Mater. Sci. Mater. Electron. 2012, 23, 1342.

[124] C. Zhao , W. Wu , H. Wang , J. Wu , J. Appl. Phys. 2016, 119, 024108.

[125] C. Zhou , W. Liu , D. Xue , X. Ren , H. Bao , J. Gao , L. Zhang , Appl. Phys. Lett. 2012, 100, 222910.

[126] D. Wang , Z. Jiang , B. Yang , S. Zhang , M. Zhang , F. Guo , W. Cao , J. Am. Ceram. Soc. 2014, 97, 3244.

[127] X. Chao , Z. Wang , Y. Tian , Y. Zhou , Z. Yang , Mater. Res. Bull. 2015, 66, 16.

[128] Y. Tian , X. Chao , L. Wei , P. Liang , Z. Yang , J. Appl. Phys. 2013, 113, 184107.

[129] Y. Tian , L. Wei , X. Chao , Z. Liu , Z. Yang , J. Am. Ceram. Soc. 2013, 96, 496.

[130] Y. Cui , X. Liu , M. Jiang , X. Zhao , X. Shan , W. Li , C. Yuan , C. Zhou , Ceram. Int. 2012, 38, 4761.

[131] Z. Zhao , X. Li , H. Ji , Y. Dai , T. Li , J. Alloys Compd. 2015, 637, 291.

[132] S. Su , R. Zuo , S. Lu , Z. Xu , X. Wang , L. Li , Curr. Appl. Phys. 2011, 11, S120.

[133] J. P. Praveen , T. Karthik , A. R. James , E. Chandrakala , S. Asthana , D. Das , J. Eur. Ceram. Soc 2015, 35, 1785.

[134] W. G. J. Holterman , An Introduction to Piezoelectric Materials and Applications, Stichting Applied Piezo, Apeldoorn, the Netherlands 2013.

[135] T. E. Hooper , J. I. Roscow , A. Mathieson , H. Khanbareh , A. J. Goetzee‐Barral , A. J. Bell , J. Eur. Ceram. Soc. 2021, 41, 6115.

[136] J. U. Rahman , A. Hussain , A. Maqbool , T. K. Song , W. J. Kim , S. S. Kim , M. H. Kim , Curr. Appl. Phys. 2014, 14, 331.

[137] Y. Hiruma , H. Nagata , T. Takenaka , Appl. Phys. Lett. 2009, 95, 052903.

[138] Y. Hiruma , H. Nagata , T. Takenaka , Jpn. J. Appl. Phys. 2009, 48, 09KC08.

[139] H.‐L. Li , Q. Liu , J.‐J. Zhou , K. Wang , J.‐F. Li , H. Liu , J.‐Z. Fang , J. Eur. Ceram. Soc. 2016, 36, 2849.

[140] J. Chen , Y. Wang , Y. Zhang , Y. Yang , R. Jin , J. Eur. Ceram. Soc. 2017, 37, 2365.

[141] R. A. Malik , J.‐K. Kang , A. Hussain , C.‐W. Ahn , H.‐S. Han , J.‐S. Lee , Appl. Phys. Express 2014, 7, 061502.

[142] S. H. Choy , X. X. Wang , H. L. W. Chan , C. L. Choy , Appl. Phys. A 2007, 89, 775.

[143] G. L. Yuan , S. Wing Or , H. Lai Wa Chan , J. Phys. D: Appl. Phys. 2007, 40, 1196.

[144] A. Galotta , V. M. Sglavo , J. Eur. Ceram. Soc 2021, 41, 1.

[145] T. Ibn‐Mohammed , C. A. Randall , K. B. Mustapha , J. Guo , J. Walker , S. Berbano , S. C. L. Koh , D. Wang , D. C. Sinclair , I. M. Reaney , J. Eur. Ceram. Soc. 2019, 39, 5213.

[146] T. Siponkoski , M. Nelo , N. Ilonen , J. Juuti , H. Jantunen , Composites, Part B 2022, 229, 109486.

[147] J. Guo , R. Floyd , S. Lowum , J.‐P. Maria , T. Herisson de Beauvoir , J.‐H. Seo , C. A. Randall , Annu. Rev. Mater. Res. 2019, 49, 275.

[148] C. Vakifahmetoglu , L. Karacasulu , Curr. Opin. Solid State Mater. Sci. 2020, 24, 100807.

[149] Y. Yu , T. Zheng , N. Zhang , J. Wu , IEEE Trans. Ultrason. Ferroelectr. Freq. Control 2022, 69, 3003. 35171771 10.1109/TUFFC.2022.3152412

[150] C. A. Randall , J. Guo , A. Baker , M. Lanagan , H. Guo , US 2017/0088471 A1, 2017.

[151] H. Guo , A. Baker , J. Guo , C. A. Randall , J. Am. Ceram. Soc. 2016, 99, 3489.

[152] J. G. Pereira da Silva , M. Bram , A. M. Laptev , J. Gonzalez‐Julian , Q. Ma , F. Tietz , O. Guillon , J. Eur. Ceram. Soc. 2019, 39, 2697.

[153] S. Grasso , M. Biesuz , L. Zoli , G. Taveri , A. I. Duff , D. Ke , A. Jiang , M. J. Reece , Adv. Appl. Ceram. 2020, 119, 115.

[154] J.‐P. Maria , X. Kang , R. D. Floyd , E. C. Dickey , H. Guo , J. Guo , A. Baker , S. Funihashi , C. A. Randall , J. Mater. Res. 2017, 32, 3205.

[155] R. Boston , J. Guo , S. Funahashi , A. L. Baker , I. M. Reaney , C. A. Randall , RSC Adv. 2018, 8, 20372. 35541645 10.1039/c8ra03072c PMC9080801

[156] J. Varghese , N. Joseph , H. Jantunen , in Encyclopedia of Materials: Technical Ceramics and Glasses, (Ed: M. Pomeroy ), Elsevier, Oxford 2021, pp. 437–451.

[157] J. Varghese , T. Siponkoski , M. Sobocinski , T. Vahera , H. Jantunen , ACS Appl. Mater. Interfaces 2018, 10, 11048. 29513520 10.1021/acsami.8b00978

[158] M. T. Sebastian , H. Jantunen , in Microwave Materials and Applications (Eds: M. T. Sebastian , R. Ubic , H. Jantunen ), Wiley, New York 2017, pp. 355–425.

[159] M. T. Sebastian , H. Jantunen , Int. Mater. Rev 2008, 53, 57.

[160] D. Jurków , T. Maeder , A. Dąbrowski , M. S. Zarnik , D. Belavič , H. Bartsch , J. Müller , Sens. Actuators, A 2015, 233, 125.

[161] S. Pang , Y. Wang , Y. Yang , J. Zhang , Q. Hu , J. Shi , X. Li , J. Am. Ceram. Soc. 2022, 105, 3438.

[162] C.‐H. Pei , M.‐L. Chen , K.‐H. Chen , C.‐M. C. , Sens. Mater. 2017, 29, 411.

[163] M. Saleem , I.‐S. Kim , M.‐S. Kim , B.‐K. Koo , S.‐J. Jeong , J. Electroceram. 2018, 40, 88.

[164] A. E. Gurdal , S. Tuncdemir , K. Uchino , C. A. Randall , Mater. Des 2017, 132, 512.

[165] S. Dursun , A. E. Gurdal , S. Tuncdemir , C. Randall , Sens. Actuators, A 2019, 286, 4.

[166] X. Hu , T. Cao , B. Wang , Z. Wen , K. Yan , D. Wu , Ceram. Int. 2023, 49, 6119.

[167] M. Nelo , T. Siponkoski , H. Kähäri , K. Kordas , J. Juuti , H. Jantunen , J. Eur. Ceram. Soc. 2019, 39, 3301.

[168] R. Zuo , J. Rödel , R. Chen , L. Li , J. Am. Ceram. Soc. 2006, 89, 2010.

[169] J. Ma , H. Li , H. Wang , C. Lin , X. Wu , T. Lin , X. Zheng , X. Yu , J. Eur. Ceram. Soc. 2019, 39, 986.

[170] B. Deng , Y. Ma , T. Chen , H. Wang , J. Lin , C. Lin , X. Wu , C. Zhao , T. Lin , M. Gao , X. Zheng , C. Fang , J. Am. Ceram. Soc. 2022, 105, 461.

[171] B. Deng , J. Jiang , H. Li , C. Zhao , C. Lin , X. Wu , M. Gao , T. Lin , J. Am. Ceram. Soc. 2022, 105, 5774.

[172] Y. Li , W. Chen , J. Zhou , Q. Xu , H. Sun , R. Xu , Mater. Sci. Eng. B 2004, 112, 5.

[173] H. Huang , J. Tang , J. Liu , Ceram. Int. 2019, 45, 6753.

[174] G. H. Haertling , J. Am. Ceram. Soc. 1999, 82, 797.

[175] D. Wang , H. Guo , C. S. Morandi , C. A. Randall , S. Trolier‐McKinstry , APL Mater. 2018, 6, 016101.

[176] X. Gao , H. Jin , B. Xin , M. Wang , S. Dong , Z. Xu , F. Li , J. Alloys Compd. 2022, 892, 162132.

[177] R. Guo , J. I. Roscow , C. R. Bowen , H. Luo , Y. Huang , Y. Ma , K. Zhou , D. Zhang , J. Mater. Chem. A 2020, 8, 3135.

[178] N. Jayasundere , B. V Smith , J. R. Dunn , J. Appl. Phys. 1994, 76, 2993.

[179] H. Luo , J. Roscow , X. Zhou , S. Chen , X. Han , K. Zhou , D. Zhang , C. R. Bowen , J. Mater. Chem. A 2017, 5, 7091.

[180] S. S. Anandakrishnan , M. Tabeshfar , M. Nelo , J. Peräntie , H. Jantunen , J. Juuti , Y. Bai , Recycling of hazardous and energy‐demanding piezoelectric ceramics using the oxide‐halide perovskite upside‐down composite method.

[181] V. Forti , C. P. Baldé , R. Kuehr , G. Bel , The Global E‐waste Monitor 2020: Quantities, flows and the circular economy potential. United Nations University (UNU)/United Nations Institute for Training and Research (UNITAR), – co‐hosted SCYCLE Programme, International Telecommunication Union (ITU) & International Solid Waste Association (ISWA). Bonn/Geneva/Rotterdam, https://ewastemonitor.info/wp-content/uploads/2020/11/GEM_2020_def_july1_low.pdf (accessed: July 2023).

[182] A New Circular Vision for Electronics‐Time for a Global Reboot. Report, World Economic Forum, 1/2019, https://www3.weforum.org/docs/WEF_A_New_Circular_Vision_for_Electronics.pdf (accessed: March 2023).

[183] Electroceramics‐Global Market Trajectory & Analytics. Global Industry Analysts, Inc report, ID: 5030055. April 2021. https://www.researchandmarkets.com/reports/5030055/electroceramics-global-strategic-business-report?utm_source=BW&utm_medium=PressRelease&utm_code=ktw3vp&utm_campaign=1415880+-+Electroceramics+-+Global+Market+Trajectory+%26+Analytics+2020-2027&utm_exec=joca220prd (accessed: July 2023).

[184] Electronic Ceramics Market by Material, Application, End‐User: Opportunity Analysis and Industry Forecast, 2020‐2027. Allied Analytics LLP report, ID: 5237544, November 2021. https://www.researchandmarkets.com/reports/5237544/electronic-ceramics-market-by-material (accessed: July 2023).

[185] M. Nelo , J. Peräntie , T. Siponkoski , J. Juuti , H. Jantunen , Appl. Mater. Today 2019, 15, 83.

[186] M. Tabeshfar , M. Nelo , S. S. Anandakrishnan , M. Siddiqui , J. Peräntie , P. Tofel , H. Jantunen , J. Juuti , Y. Bai , Oxide‐halide perovskite composites for simultaneous recycling of lead zirconate titanate piezoceramics and methylammonium lead iodide solar cells. 10.1002/smtd.202300830 38072621

[187] Y. Bai , H. Jantunen , J. Juuti , Adv. Mater. 2018, 30, 1707271. 10.1002/adma.201707271 29877037

[188] H. Birol , D. Damjanovic , N. Setter , J. Am. Ceram. Soc. 2005, 88, 1754.

[189] K. Yanagisawa , M. Nishioka , K. Ioku , N. Yamasaki , J. Mater. Sci. Lett. 1993, 12, 1073.

[190] T. Morita , Materials 2010, 3, 5236. 28883379 10.3390/ma3125236 PMC5445807

[191] M. B. Ghasemian , Q. Lin , E. Adabifiroozjaei , F. Wang , D. Chu , D. Wang , RSC Adv. 2017, 7, 15020.

[192] N. Wei , T. Karaki , T. Fujii , Jpn. J. Appl. Phys 2022, 61, SN1018.

[193] T. Morita , Y. Wagatsuma , H. Morioka , H. Funakubo , N. Setter , Y. Cho , J. Mater. Res 2004, 19, 1862.

